# Experimentally disambiguating models of sensory cue integration

**DOI:** 10.1167/jov.22.1.5

**Published:** 2022-01-12

**Authors:** Peter Scarfe

**Affiliations:** 1 Vision and Haptics Laboratory, School of Psychology and Clinical Language Sciences, University of Reading, Reading, UK

**Keywords:** sensory cue integration, Bayesian, modelling, sensory cue combination

## Abstract

Sensory cue integration is one of the primary areas in which a normative mathematical framework has been used to define the “optimal” way in which to make decisions based upon ambiguous sensory information and compare these predictions to behavior. The conclusion from such studies is that sensory cues are integrated in a statistically optimal fashion. However, numerous alternative computational frameworks exist by which sensory cues could be integrated, many of which could be described as “optimal” based on different criteria. Existing studies rarely assess the evidence relative to different candidate models, resulting in an inability to conclude that sensory cues are integrated according to the experimenter's preferred framework. The aims of the present paper are to summarize and highlight the implicit assumptions rarely acknowledged in testing models of sensory cue integration, as well as to introduce an unbiased and principled method by which to determine, for a given experimental design, the probability with which a population of observers behaving in accordance with one model of sensory integration can be distinguished from the predictions of a set of alternative models.

## Introduction

### Integrating sensory information

Humans have access to a rich array of sensory data from both within and between modalities upon which to base perceptual estimates and motor actions. These data are treated as consisting of quasi-independent sensory “cues.” Given a set of cues, the question then becomes how information is integrated to generate a robust percept of the world (Ernst & Bulthoff, 2004). Mathematically, there are multiple ways in which this could occur (Jones, 2016; Tassinari & Domini, 2008; Trommershauser, Körding, & Landy, 2011), however, currently the most popular theory is the minimum variance unbiased estimator model (MVUE). MVUE forms part of broader computation frameworks, such as modified weak fusion (MWF; Landy, Maloney, Johnston, & Young, 1995) and is related to Bayesian models of sensory perception (Knill & Richards, 1996). Indeed, sensory cue integration has been described as the “… poster child for Bayesian inference in the nervous system” (Beierholm, Shams, Körding, & Ma, 2009, p. 1).

In MVUE, given two cues, ${\hat{S}}_{A}$ and ${\hat{S}}_{B}$, each corrupted by statistically independent zero-mean Gaussian noise with variances $\sigma_{A}^{2}$ and $\sigma_{B}^{2}$, it can be shown, given some additional assumptions, that the integrated cues estimate, ${\hat{S}}_{C}$, is given by a simple weighted average (for derivations see Cochran, 1937; Oruc, Maloney, & Landy, 2003).

(1) $${\hat{S}}_{C}=w_{A}{\hat{S}}_{A}+w_{B}{\hat{S}}_{B}$$

The weights are determined by the relativity reliability of the cues ($r_{A}=1/\sigma_{A}^{2}$ and $r_{B}=1/\sigma_{B}^{2}$) such that *w_A_* = *r_A_*/(*r_A_* + *r_B_*) and *w_B_* = *r_B_*/(*r_A_* + *r_B_*) and the standard deviation (sigma) of the Gaussian probability density function representing the integrated cues estimator is given by

(2) $$\sigma_{C}=\sqrt{\frac{\sigma_{A}^{2}*\sigma_{B}^{2}}{\sigma_{A}^{2}+\sigma_{B}^{2}}}$$

**Figure 1.**
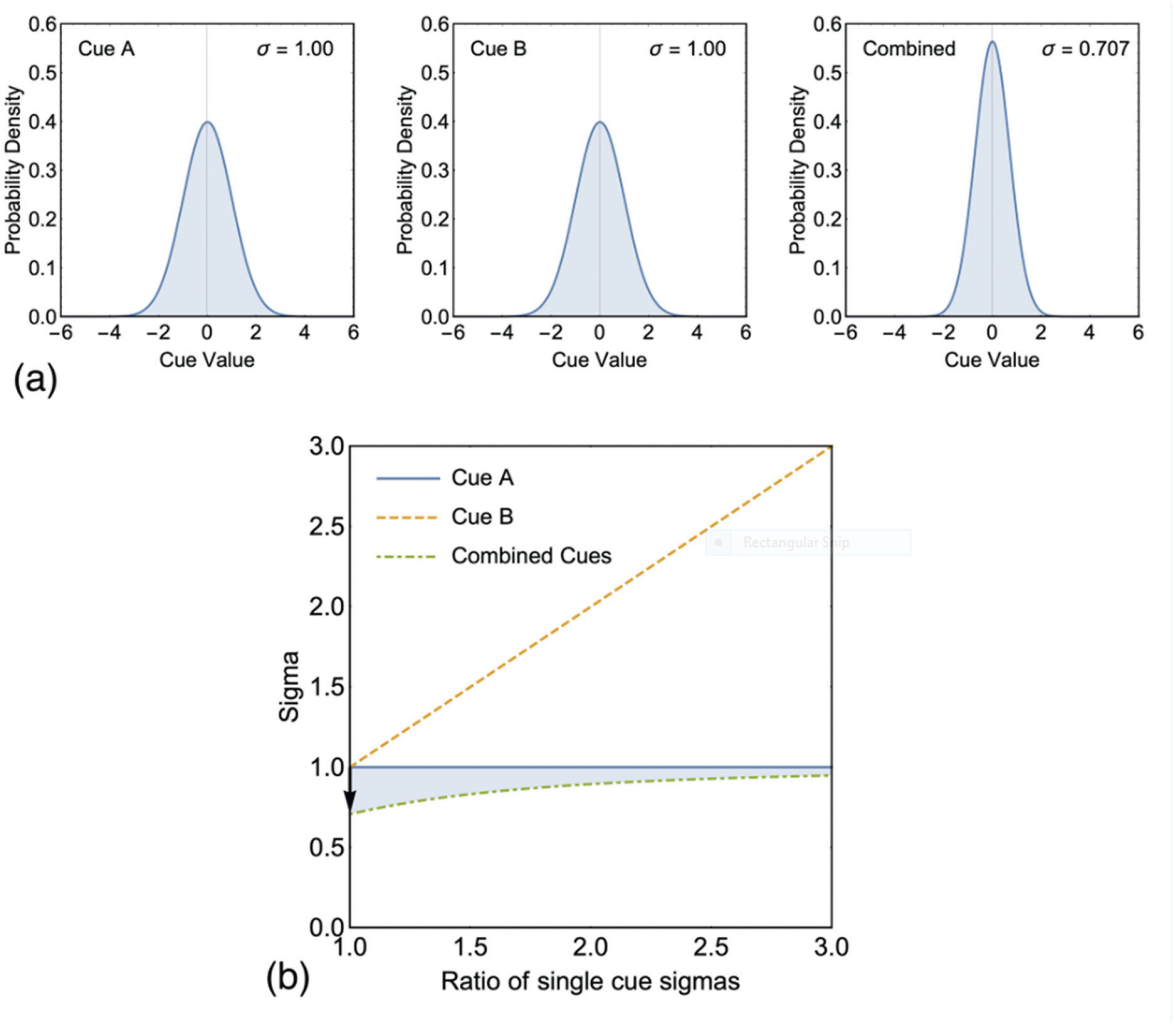
(**a**) Shows a hypothetical example of integrating two cues (A and B) with identical reliabilities (for both cues σ = 1). In this instance an observer would maximally benefit from integrating cues in accordance with Equation 2 and obtain a $\sqrt{2}$ reduction in sigma. (**b**) Plots single cue sigmas and the integrated cues sigma associated with the two cues for a range of sigma ratios. A sigma ratio of one indicates that the two cues are equally reliable (as in **a**). A value greater than one indicates that cue B is more variable than cue A. The shaded region shows the increased precision afforded by integrating cues in accordance with Equation 2. The black arrow shows the maximally achievable increase in precision shown in **a**.

The key benefit of integrating cues in this way is that the sigma of the integrated cues estimator is always less than or equal to the sigma of the most reliable of the individual sensory cues. As a result, MVUE is often terms “optimal cue integration” (Rohde, van Dam, & Ernst, 2016). Whereas there are clearly multiple benefits of combining and integrating sensory information (Ernst & Bulthoff, 2004), MVUE posits that the optimizing criteria of sensory integration is to maximize the precision of the integrated cues sensory estimate. The maximum reduction in sigma (increase in precision) is achieved when the two cues are equally reliable (Figure 1). As the reliability of the cues becomes unbalanced, the increase in precision rapidly diminishes (Figure 1b). Therefore, in terms of sensitivity, for an organism to do better than simply choosing the most reliable of the two cues, the cues must be approximately matched in reliability.

Numerous studies purport to show that humans combine cues in accordance with MVUE (Burge, Girshick, Banks, 2010; Ernst, 2006; Ernst & Banks, 2002; Gepshtein, Burge, Ernst, & Banks, 2005; Girshick & Banks, 2009; Glennerster, Tcheang, Gilson, Fitzgibbon, & Parker, 2006; Helbig & Ernst, 2007; Hillis, Ernst, Banks, & Landy, 2002; Hillis, Watt, Landy, & Banks, 2004; Johnston, Cumming, & Landy, 1994; Johnston, Cumming, & Parker, 1993; Knill & Saunders, 2003; Lovell, Bloj, & Harris, 2012; Saunders & Chen, 2015; Scarfe & Hibbard, 2011; Svarverud, Gilson, & Glennerster, 2010; Watt, Akeley, Ernst, & Banks, 2005). However, for the MVUE model to apply, several assumptions need to be met. Although acknowledged across the literature, these assumptions are rarely mentioned in experimental tests of MVUE and are often simply assumed to have been met.

### Assumptions of MVUE

To be integrated in MVUE, cues must be in common units and these modeled units need to be equivalent to the units that the observer is using to make perceptual estimates. For example, if an observer is judging surface “slant” from “disparity” and “texture” cues (Hillis et al., 2004), however information from these cues is processed in the brain, it has to result in a measure of “slant” in the same units (e.g. degrees or radians). If it does not, Equation 1 is meaningless as it will be averaging a property in two different units. As a result, frameworks which incorporate MVUE as a core component, such as MWF, have a “cue promotion” stage prior to averaging where cues are promoted so as to be in common units (Landy et al., 1995). Cue promotion, although critical, is rarely directly studied (although see Burge, Fowlkes, & Banks, 2010; Burge, Peterson, & Palmer, 2005). Whilst it is possible to experimentally evaluate the units an observer is using to make perceptual estimates, for example, Hillis et al. (2004) examined whether their observers were estimating “slant” from disparity, or simply doing the task based upon disparity gradient, this is rarely carried out. Typically, experimenters assume from the outset that cues are in common units and that these units are equivalent to those the observers are using to make perceptual estimates.

Additionally, in MVUE, cues are integrated regardless of their perceptual bias. By “bias” we mean a difference between (a) the perceptual estimate of a property of the world and (b) the actual physical value of that property. This has been termed “external accuracy” (Burge, Girshick, et al., 2010). Bias is a problem, in part, because there is no reason to assume that cues which are more reliable are also least biased. As a result, there are a mathematically definable range of circumstances where integrating cues in accordance with Equations 1 and 2 results in perceptual estimates which are more precise, but less accurate with respect to the world (Scarfe & Hibbard, 2011). As experimenters have no direct access to an observer's internal perceptual estimates, cues are generally assumed to be unbiased, with any bias attributed to unmodeled cue conflicts or response bias (Watt et al., 2005).

The assumption of unbiased estimates is problematic given the large reproducible perceptual biases shown in real world environments (Bradshaw, Parton, & Glennerster, 2000; Koenderink, van Doorn, Kappers, & Lappin, 2002; Koenderink, van Doorn, Kappers, & Todd, 2002; Koenderink, van Doorn, & Lappin, 2000; Wagner, 1985), and in expertly controlled experiments with computer generated stimuli (Watt et al., 2005). These biases suggest that sensory cues are not necessarily accurately calibrated with respect to the world (Adams, Banks, & van Ee, 2001; Henriques & Cressman, 2012; McLaughlin & Webster, 1967; Scarfe & Glennerster, 2014; Welch, Bridgeman, Anand, & Browman, 1993) and importantly, it has been shown that the integration of sensory cues does not lead to those same cues being accurately calibrated (Smeets, van den Dobbelsteen, de Grave, van Beers, & Brenner, 2006). As a result, it is now becoming accepted that bias in sensory estimates needs to be accounted for in models of sensory cue integration (see Ernst & Di Luca, 2011). Indeed, one can experimentally examine the effects of discrepancies between cues and model cue integration in terms of causal inference, whereby the brain evaluates the probability with which signals come from common or distinct external causes and uses this to gate cue integration (Beierholm et al., 2009; Gepshtein et al., 2005; Körding, Beierholm, Ma, Quartz, Tenenbaum, & Shams, 2007).

For Equations 1 and 2 to hold, each cue needs to be well represented by a statistically independent Gaussian probability density function (Cochran, 1937). A clear case where this does not hold is for circularly distributed variables, such as planar direction. With circularly distributed variables the von Mises distribution should be used, and equations similar to MVUE can be derived with some additional assumptions (Murray & Morgenstern, 2010). However, many studies simply assume that over the stimulus domain tested, Gaussian distributions provide a good enough approximation to the underlying von Mises distributions (Hillis et al., 2004). Similarly, when statistical independence does not hold, corrections to the MVUE equations can be derived to account for correlated noise (Oruc et al., 2003), however, in virtually all studies, statistical independence is assumed a priori or the correlation assumed to be so small that MVUE provides a valid approximation.

The final assumption we consider here is that, over the domain being investigated, the perceptual scale of the cues is linear. Perceptual scales are known to be nonlinear (Rohde et al., 2016), so the domain over which cue integration is investigated is typically restricted and assumed to be a close approximation to linear (e.g. Hillis et al., 2004). However, even in these instances, it has been claimed that in some circumstances observers may be making perceptual estimates based upon confounding cues which are nonlinear over the experimental domain and that as a result the experimental methodology used to estimate the precision of cues will misestimate the variance of the underlying estimators (Todd, Christensen, & Guckes, 2010; Todd & Thaler, 2010). This continues to be a contentious area of active debate (Saunders & Chen, 2015; Todd, 2015).

### Experimentally testing MVUE

Testing MVUE equates to seeing if the numerical predictions made by Equations 1 and 2 correspond with observer behavior. Of the two predictions, Rohde, van Dam and Ernst (2016, p. 7) describe Equation 2 as the “essential prediction” of optimal cue integration. They point out that seeing performance line with Equation 1 is “… by itself not sufficient to show that optimal integration occurs” (Rohde et al., 2016, p. 7). As a result, “(i)f one can show only a bias in the results (Equation 1) but not a reduction in noise (Equation 2), one *cannot conclude that optimal integration occurred* …” (Rohde et al., 2016, p. 10. Note: equation numbers have been changed to correspond to the equivalent equations in the current paper and italics added.).

There are two key reasons for this. First, identical predictions to Equation 1 can be made by alternative models of perceptual processing, including those in which cues are not integrated in any way. Second, testing Equation 1 by adding an experimental cue conflict through a “perturbation analysis” (Ernst & Banks, 2002; Young, Landy, & Maloney, 1993) can be severely disrupted if one or more of the perceptual estimates is biased. To demonstrate this, following on from the example above, we can experimentally add a perturbation of value Δ to the cue ${\hat{S}}_{B}$, such that

(3) $${\hat{S}}_{C}=w_{A}{\hat{S}}_{A}+w_{B}\left({\hat{S}}_{B}+\Delta \right)$$

We can then ask what value of bias β in cue ${\hat{S}}_{A}$ would be required to eradicate any evidence of optimal cue integration

(4) $${\hat{S}}_{C}=w_{A}\left({\hat{S}}_{A}+\beta \right)+w_{B}\left({\hat{S}}_{B}+\Delta \right)$$

Recognizing that *w_A_* + *w_B_* = 1 and solving for β gives

(5) $$\beta =-\Delta \frac{\sigma_{A}^{2}}{\sigma_{B}^{2}}$$

Thus, in a perturbation analysis, all signs of optimal cue integration can be eliminated if one or more of the perceptual estimates are biased and this depends on the relative reliability of the cues and the magnitude of the perturbation. Rohde et al. (2016) recommend that Δ be 1 to 1.5 (and no larger than 2) just noticeable differences (JNDs), where a JND is given by $\sigma \sqrt{2}$, so as not to elicit cue veto (Landy et al., 1995). Therefore, whereas the ratio of cue variances being exactly that shown in Equation 5 is unlikely, cues only need to be biased by a small amount to significantly interfere determining cue weights through a perturbation analysis. This, coupled with identical predictions to Equation 1 being made by alternative models, is why Equation 2 is seen as the essential prediction of MVUE (Rohde et al., 2016).

### Comparing models of cue integration

Although MVUE is the most widely accepted model of cue integration, there are numerous alternatives, many of which take into account the reliability of sensory cues (Arnold, Petrie, Murray, & Johnston, 2019; Domini & Caudek, 2009; Jones, 2016; Rosas & Wichmann, 2011; Tassinari & Domini, 2008). Much of the difference between models comes down to the computational architecture of the underlying system (Beierholm et al., 2009; Körding et al., 2007; Trommershauser et al., 2011). Therefore, as within any area of science, the question comes down to designing experiments which can distinguish between competing models. However, until recently, very few papers compared the predictions of MVUE to alternative models in any rigorous fashion (for exceptions see Acerbi, Dokka, Angelaki, & Ma, 2018; de Winkel, Katliar, Diers, & Bulthoff, 2018; Lovell et al., 2012). This has been recognized as a clear weakness in claiming that cues are integrated in accordance with MVUE (Arnold et al., 2019).

An additional problem is that readers are often required to judge the fit of the data to MVUE “by eye,” without any accompanying statistics detailing the fit of the model to the data (e.g. Ernst & Banks, 2002; Hillis et al., 2004). A recent review has suggested that the adherence to MVUE can be assessed visually and has provided a visual taxonomy of “optimal,” “suboptimal,” “ambiguous,” “near optimal,” and “supra-optimal” performance (Rohde et al., 2016, p. 23). This visual taxonomy, based on judging the fit to the predictions of MVUE from visual inspection of (1) the data, (2) the error bars around the data, and (3) the predictions of MVUE, has started to be used by researchers to assess the “optimality” of experimental data (Negen, Wen, Thaler, & Nardini, 2018).

A visual taxonomy is problematic for many reasons. First, across a range of disciplines, including psychology, behavioral neuroscience, and medicine, leading researchers have been shown to have fundamental and severe misconceptions about how error bars relate to statistical significance and how they can be used to support statistical inferences from data (Belia, Fidler, Williams, & Cumming, 2005; Cumming, Fidler, & Vaux, 2007). Second, as will be seen, alternative models of cue integration provide highly correlated predictions with one another. Therefore, “eyeballing” the fit to a single model based on visual inspection is likely to lead to fundamental mistakes in inferring the extent to which a given model fits the data, especially when the literature in this area tends to be focused upon verification, but not necessarily falsification (Rosas & Wichmann, 2011). Finally, there are techniques which can be easily used to assess the fit of a set of candidate models to data in a far more objective way.

### Outline of the current study

Here, we present a technique consisting of simulating end-to-end experiments (behavior of observers in an experiment, fitting of psychometric functions, estimation of parameters from data, and final statistical analysis), which can be used to determine the probability with which a population of observers behaving in accordance with one model of sensory cue integration can distinguished from the predictions of a set of alternative models. Given its ubiquity, we focus on the extent to which the predictions of MVUE can be distinguished from two popular alternative models, (a) choose the cue with the minimum sigma (MS), and (b) probabilistic cue switching (PCS). There are numerous other models which could have been chosen for comparison (Jones, 2016), however, these two models have the benefits of (1) being conceptually similar to MVUE, (2) require experimental estimation of the same parameters, and (3) are reducible to comparably simple equations. They have also been compared to the predictions of MVUE in previous papers.

## Methods and results

### Correlated predictions of alternative models

**Figure 2.**
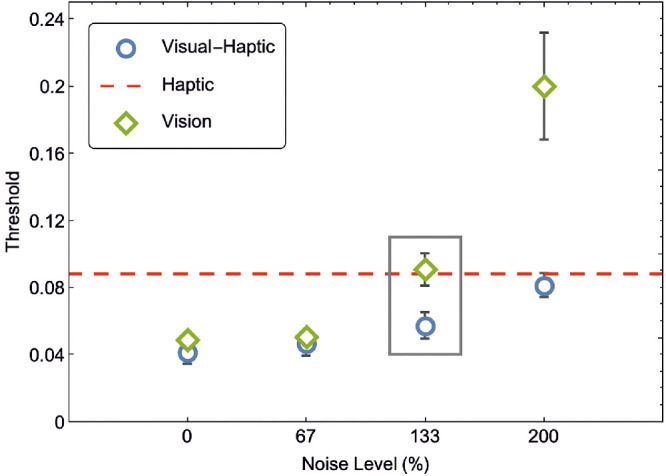
Replot of the threshold data from Ernst and Banks (2002) Figure 3d (see Supplementary Figure S1 for details). The threshold is defined as the difference between the 84% and 50% point of the underlying psychometric cumulative Gaussian function. Thus, smaller thresholds represent more precise perceptual estimates. Thresholds are plotted against the percentage of noise in the visual modality stimulus (see Ernst & Banks, 2002 for full details). The only datapoint which can clearly distinguish MVUE from MS is the 133% noise level stimulus (grey rectangle).

When choosing the cue with the MS, the sigma of the integrated cues estimator is simply the sigma of the most reliable cue. Therefore, when the reliabilities of the two cues are imbalanced MS provides highly similar predictions to MVUE. This can be seen in Figure 2 where we re-plot discrimination thresholds for the visual, haptic, and integrated cue estimators from Ernst and Banks (2002) (see Supplementary Figure S1). For the 0, 67, and 200% noise conditions, the discrimination threshold for the integrated cues estimator is visually indistinguishable from the threshold of the most reliable of the individual cues (visual or haptic). Thus, the only condition in this paper where MS and MVUE make clearly different predictions is the 133% noise condition where the reliabilities of the two cues are nearly identical (grey rectangle).

Although one could argue the 133% datapoints distinguish models, there are a few complications in making this inference, as there are no statistics reported to assess this difference and it is not stated what the error-bars (calculated over four observers) show. As a result, the reduction in sigma for the 133% condition is a visual judgment on the part of the reader. As detailed above, leading researchers have been shown to have fundamental misconceptions about visual judgments about statistical significance from visual inspection (Belia et al., 2005; Cumming et al., 2007). Although attaching a “*p* value” to a result is clearly not the only way in which to make inferences from data (and can be highly problematic; Kruschke, 2010, 2011). It is acknowledged that more rigorous methodologies are required to distinguish between competing cue integration models (Acerbi et al., 2018).

PCS (Byrne & Henriques, 2013; de Winkel et al., 2018; Nardini, Jones, Bedford, & Braddick, 2008; Serwe, Drewing, & Trommershauser, 2009) proposes that observers do not integrate cues to form a single perceptual estimate, rather, they use a single cue at a given time and switch between cues with the probabilities *p_A_* and *p_B_* (where *p_A_* + *p_B_* = 1). The mean and sigma of the integrated cues estimator is given by

(6) $${\hat{S}}_{C}=p_{A}{\hat{S}}_{A}+p_{B}{\hat{S}}_{B}$$

and

(7) $$\;\;\;\begin{array}{c} \sigma_{C}=\sqrt{\frac{\sigma_{A}^{2}\sigma_{B}^{2}\left({\hat{S}}_{A}^{2}-2*{\hat{S}}_{A}{\hat{S}}_{B}+{\hat{S}}_{B}^{2}+2*\left(\sigma_{A}^{2}+\sigma_{B}^{2}\right)\right)}{{\left(\sigma_{A}^{2}+\sigma_{B}^{2}\right)}^{2}}} \end{array}$$

When ${\hat{S}}_{A}={\hat{S}}_{B}$, Equation 7 simplifies further to

(8) $$\sigma_{C}=\sqrt{2}\sqrt{\frac{\sigma_{A}^{2}*\sigma_{B}^{2}}{\sigma_{A}^{2}+\sigma_{B}^{2}}}$$

The similarities between (Equations 6) and (1), and (Equations 8) and (2) are clear. Note that (Equations 6) and (1) provide identical predictions when *p_A_* = *w_A_* and *p_B_* = *w_B_*. In other words, for the mean of the integrated cues estimator, a model in which cues are not integrated and instead used completely independently can produce identical predictions to MVUE. Throughout the paper where PCS is modeled, we have set *p_A_* = *w_A_* and *p_B_* = *w_B_*, so as to be consistent with previous research where these parameters are estimated and modelled (e.g. Byrne & Henriques, 2013). However, it is true that in reality *p_A_*, *p_B_*, *w_A_*, and *w_B_* could be determined by more than simply the relative reliability of cues as measured with a 2AFC forced choice experiment that experimenters typically adopt to estimate these parameters (see Jacobs, 2002 for an extended discussion).

**Figure 3.**
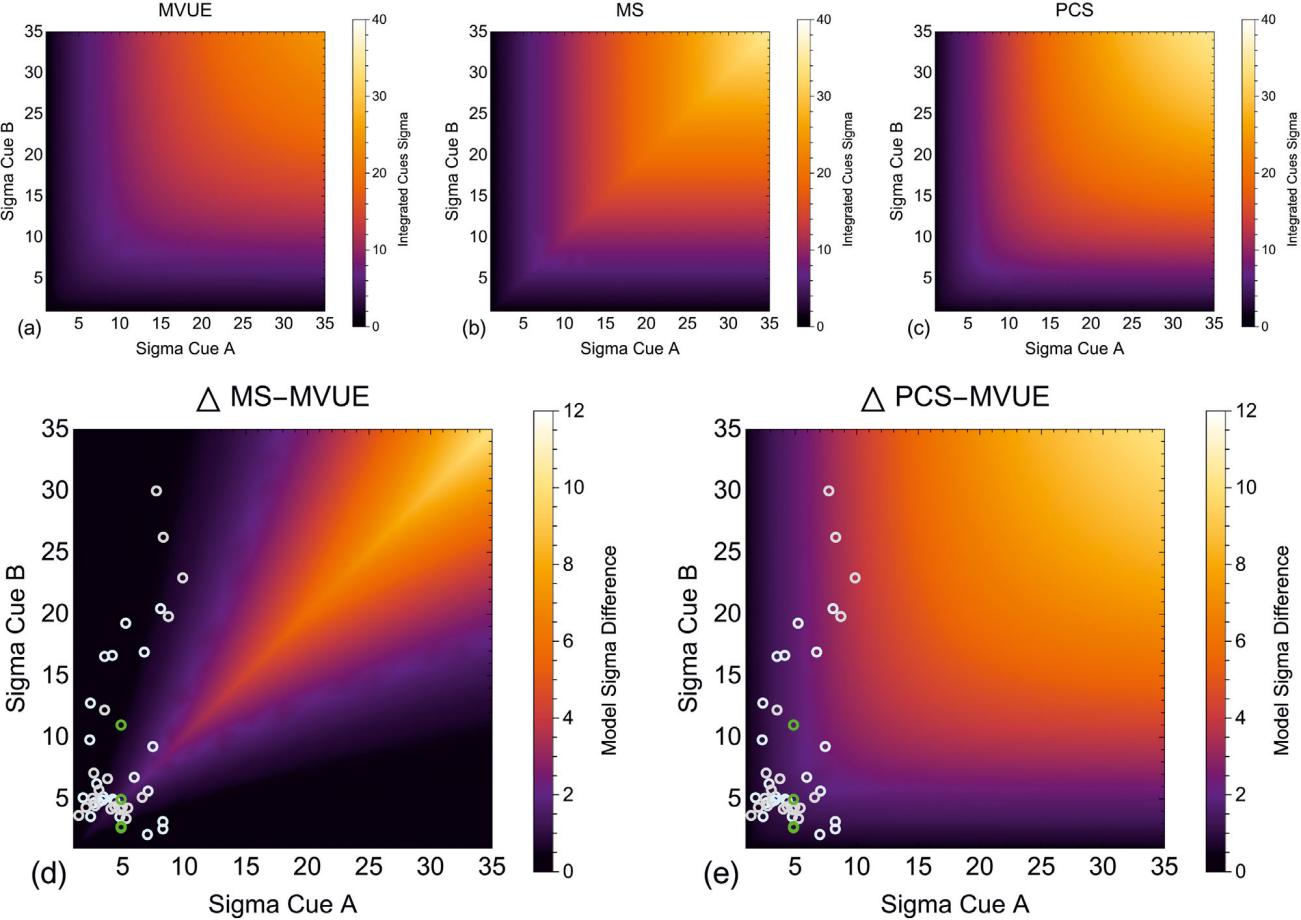
Shows the integrated cues sigma, for a range of two-cue sigma values under our three models of cue integration, (**a**) MVUE (Equation 2), (**b**) MS, and (**c**) PCS (Equation 8). (**d**) Plots the difference in integrated cues sigma predicted by MS versus MVUE and (**e**) PCS versus MVUE. In these two plots green symbols show the sigma values from Figure 3d in Ernst and Banks (2002) for the perception of object height. Cyan and grey symbols show sigma values from Figure 11 in Hillis, Watt, Landy and Banks (2004) for the perception of surface slant (cyan symbols observer JMH and grey symbols observer ACD).

Figures 3a to c plots the predictions for the sigma of the integrated cues estimator under MVUE, MS, and PCS for a range of cue relative reliabilities. For PCS, the two cues have been set to have the same mean (i.e. Equation 8). The three models provide highly correlated predictions. Figures 3d and 3e take the difference in the predictions of the models. MS and PCS both provide maximally different predictions from MVUE when the sigma of the individual cues is identical (positive diagonal), and the absolute magnitude of this difference increases with the sigma of the two cues (compare the bottom left to top right in each plot). Also plotted are data points from two of the most widely cited papers on optimal cue integration, Ernst and Banks (2002) and Hillis, Watt, Landy and Banks (2004) (method as in S1). Whereas some of these datapoints lay near the positive diagonal, many datapoints fall into areas of the parameter space, which poorly distinguished MVUE from MS and PCS.

The correlated predictions of models of cue integration are a known problem (Arnold et al., 2019; de Winkel et al., 2018). Indeed, the explicit aim of some of the key earlier studies in the area was to distinguish between models. For example, Ernst and Banks (2002) aimed to distinguish between MVUE and the prevailing wisdom that vision dominated haptics when the modalities were in conflict (for an extended discussion see Rohde et al., 2016). Subsequent studies have focused on examining areas of the parameter space which maximally distinguish between models, such as MVUE and MS (e.g. Takahashi, Diedrichsen, & Watt, 2009) and more rigorous model comparison approaches have been adopted (Acerbi et al., 2018; Lovell et al., 2012), however, this is not the norm. As a result, if the aim is to distinguish between models, there are many things that could be improved upon in this area.

### General methods

All simulations were carried out in MATLAB (2020a; MathWorks, Natick, MA, USA) on an 8-Core Intel Core i9 processor in a MacBook Pro running macOS 10.15. The simulations reported were computationally expensive, so where possible they were distributed over the computer's CPU cores using MATLAB's Parallel Processing Toolbox. The Palamedes toolbox was used to parametrically simulate observers and fit psychometric functions (Kingdom & Prins, 2010, 2016; Prins & Kingdom, 2009, 2018).

## Simulation Set 1: Effects of relative reliability and number of observers in an experiment on distinguishing between candidate models

### Methods

#### Simulating observers

Observers were assumed to have access to two cues (${\hat{S}}_{A}$ and ${\hat{S}}_{B}$) from which to make an integrated cues perceptual estimate (${\hat{S}}_{C}$) about a property of the world. The mean of the cues prior to any perturbation was the same (55 mm as in Ernst and Banks (2002)). Cue A always had the same sigma (σ_*A*_ = 4.86), which is approximately that of the haptic cue in Ernst and Banks (2002). Cue B had a sigma given by σ_*B*_ = σ_*A*_*r* where *r* varied between 1 and 4 in 27 linearly spaced steps. Although it has been suggested that to test for optimal cue integration, the sigma ratio should be no larger than 2 (Rohde et al., 2016, p. 15), it is evident that experimenters go beyond this reliability ratio (see Figure 3). Thus, in the simulations presented we go beyond a ratio of 2 to be consistent with the experimental literature. For each reliability ratio, we simulated experiments where there were 4 through 30 (in steps of 1) participants. Cue integration experiments normally have few observers per experiment, but a substantial amount of data collected per observer (Rohde et al., 2016). For example, Ernst and Banks (2002) and Hillis, Watt, Landy and Banks (2004), each used four observers. Our highest observer number therefore represents an upper limit to the observers one might reasonably expect to see in a cue integration study.

The procedure described was repeated for three levels of cue conflict and four data collection regimes. The simulated conflicts, Δ, were 0, 3, and 6 mm (as in Ernst & Banks, 2002). Conflicts were added by perturbing each cue by opposite amounts equal to half of the total cue conflict, that is *S_A_* = 55 + Δ/2 and *S_B_* = 55 − Δ/2. Estimated from the data of Ernst and Banks (2002), the above zero conflicts represented approximately 0.8 and 0.4 JNDs, which is around the recommended magnitude of cue conflict to use in a perturbation analysis (Rohde et al., 2016). In Ernst and Banks (2002) there were conditions with equal and opposite cue conflicts applied in order avoid perceptual adaptation. We did not replicate this here as our simulated observers have no mechanisms of adaptation.

We simulated performance and estimated three psychometric functions for each observer in each experiment. Two single cue functions, corresponding to the stage at which an experimenter estimates single cue sensitivities and an integrated cues condition where observers behaved in accordance with MVUE. Observers were simulated with a Cumulative Gaussian function consistent with the underlying mean and sigma of the Gaussian probability density function representing the internal estimator. Functions were sampled with the method of constant stimuli, under four data collection regimes. The method of constant stimuli was selected as this is the most widely used procedure for estimating a psychometric function. Rohde, van Dam, and Ernst describe it as “… the simplest and least biased method to measure a complete psychometric function” (p.15).

The sampling space over which the psychometric function was estimated was set to 20 mm (based upon that of Ernst and Banks (2002)) and was always centered upon the true mean of the psychometric function. Centering on the true mean represents a best-case scenario for estimating the (normally unknown) function parameters. In terms of sampling density, Rohde et al. (2016) conclude that “(i)n most cases a fixed set of seven or nine comparison stimuli can be identified that suits most observers” (p. 14). Here, we adopt the upper of these suggestions and spaced the stimuli linearly across the sampling range.

It is an open question how many times each stimulus should be sampled. Rohde, van Dam, and Ernst (2016) suggest that when the mean and slope of the function need to be estimated around 150 trials should be used. Kingdom and Prins (2010) suggest that “400 trials is a reasonable number to aim for when one wants to estimate both the threshold and slope of the PF” (p. 57. PF, being Psychometric Function). In a simulation study, Wichmann and Hill (2001a) found that for some of their simulated sampling schemes, 120 samples in total per function was often “… too small a number of trials to be able to obtain reliable estimates of thresholds and slopes …” (p. 1302). Therefore, here, in separate simulations, we examined sampling with 10, 25, 40, and 55 trials per stimulus level, giving us 90, 225, 360, and 495 trials per function.

Piloting showed that throughout the present study, these parameters resulted in well fit psychometric functions (see S2 and the criteria adopted for rejected functions detailed below). Although not as widely used, we could have used an adaptive method by which to sample the psychometric function (Leek, 2001). We opted not to do so (a) to be consistent with the most widely used psychophysical methods used in the literature (Rohde et al., 2016), (b) to avoid decisions related to which method to use (Kingdom & Prins, 2016; Kontsevich & Tyler, 1999; Pentland, 1980; Prins, 2013; Watson, 2017; Watson & Pelli, 1983), and (c) to avoid the issue to the adaptive method getting “stuck” in uninformative regions of the parameter space (see Prins, 2013). Additionally, adaptive procedures are often used when the experimenter does not know the parameters of the underlying functions, which was not the case here.

#### Fitting functions

With the above permutations, for the first set of simulations, we simulated 27 (reliability ratios) × 27 (number of observers / experiment) × 4 (data collection regimes) × 3 (cue conflicts) × 100 (repetitions of experiment) = 874,800 experiments. In total, these experiments contained 14,871,600 simulated observers. Simulated data were fit with Cumulative Gaussian functions by maximum likelihood using the Palamedes toolbox. Although other fitting methods could be used, for example, fitting based on a Bayesian criterion (Kingdom & Prins, 2010; Kuss, Jakel, & Wichmann, 2005; Schütt, Harmeling, Macke, & Wichmann, 2016), fitting by maximum likelihood is currently the most widely used technique in the literature (Kingdom & Prins, 2010; Wichmann & Hill, 2001a; Wichmann & Hill, 2001b). For all simulations, we modeled observers as making zero lapses, so when fitting functions, we fixed the lapse rate to be zero (Prins, 2012; Wichmann & Hill, 2001a, 2001b).

For our simulated observers each perceptual judgment is statistically independent of all others. Therefore, there was no need here to correct for “non-stationarity” in observers’ behavior during the fitting process (Fründ, Haenel, & Wichmann, 2011; Schütt et al., 2016). This is clearly not the case in an experimental setting, where there is clear evidence that the decisions made by an observer on a given trial can be influenced by previous decisions the observer has made (Fischer & Whitney, 2014; Fründ et al., 2011; Kiyonaga, Scimeca, Bliss, & Whitney, 2017; Lages & Jaworska, 2012; Liberman, Fischer, & Whitney, 2014; Liberman, Manassi, & Whitney, 2018; Liberman, Zhang, & Whitney, 2016; Xia, Leib, & Whitney, 2016).

The mean and standard deviation of the fitted functions were taken as the experimental estimates of the observers’ true internal parameters. In cases where a function could not be fit due to the simulated data being (1) at or around chance performance across all stimulus levels, or (2) a step function, the data for that simulated observer were removed from the analysis (see also S2). Overall, this represented 0.047% of the data. The removed observers for each number of “trials per psychometric function” were: 90 trials/function = 0.183%, 225 trials/function = 0.0025%, 360 trials/function = 0.00006%, and 495 trials/function = 0%. An alternative analysis where poorly fit functions are replaced by a newly simulated observer results in identical conclusions being made throughout the paper.

#### Comparing the data to alternative models

For each simulated observer, the mean and sigma of the single cue function with the lowest sigma was taken as the experimental prediction for MS. For PCS, the mean and sigma of the single cue functions were entered into Equations 6 and 7 to provide predictions for the integrated cues function, with *p_A_* = *w_A_* and *p_B_* = *w_B_*. Dividing sigma's by $\sqrt{2}$, as in a typical two interval forced choice procedure (Green & Swets, 1974), was not needed as the functions were parametrically simulated. For each simulated experiment, the MVUE data were entered into a one-sample within-subjects *t*-test and compared to the point predictions of MS and PCS. The mean value of the alternative model prediction across observers was taken as the point prediction for each model.

Our simulated data are measured on a ratio scale and all observations are independent of one another, however, we do not know that the data are normally distributed and that parametric statistical tests are appropriate. Examining the literature, it is clear that where statistical tests are run, data normality is typically not reported, but parametric statistical tests are used. Indeed, given the small number of observers in a cue integration experiment, it would be difficult to reliably estimate the normality of the data. Adopting parametric tests was therefore considered a reasonable choice (using a nonparametric Wilcoxon signed rank test results in the same conclusions being made throughout). We adopted the standard (but arbitrary) *p* < 0.05 level for “statistical significance”.

#### Group analysis: Integrated cues sensitivity

**Figure 4.**
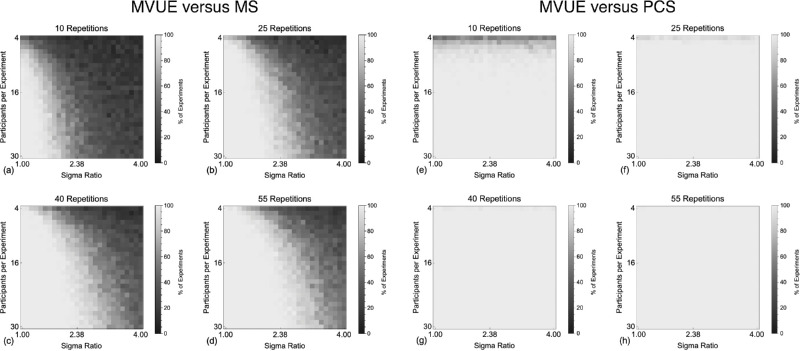
Shows the percentage of experiments in which the sigmas of the Cumulative Gaussian functions fit to our simulated population of MVUE observers could be statistically distinguished from the experimentally derived prediction of MS (**a–d**) and PCS (**e–h**). Pixels in the images show this percentage (as calculated across 100 simulated experiments) for a given sigma ratio and number of participants. This is shown for **a** and **e** 10, **b** and **f** 25, **c** and **g** 40, and **d** and **h** 55, simulated trials per stimulus level on the psychometric function.

First, we examined the extent to which MVUE, MS, and PCS can be distinguished based on the sensitivity of the integrated cues estimator. In Figure 4 the shading of each pixel represents the percentage of simulated experiments in which the results of a population of observers behaving in accordance with MVUE could be statistically distinguished from the numerical predictions of MS and PCS. Consistent with the correlated predictions of candidate models (see Figure 3), as the sigma of the individual cues becomes unbalanced it becomes progressively more difficult to experimentally distinguish between MVUE and MS. This is especially apparent with the low number of observers that characterize typical cue integration experiments. As would be expected, when more data are collected per function, models can be more easily distinguished. MVUE observers can be easily distinguished from the sigma value predicted by PCS across all sigma ratios and data collection regimes, as would be expected from Equations 2 and 8.

**Figure 5.**
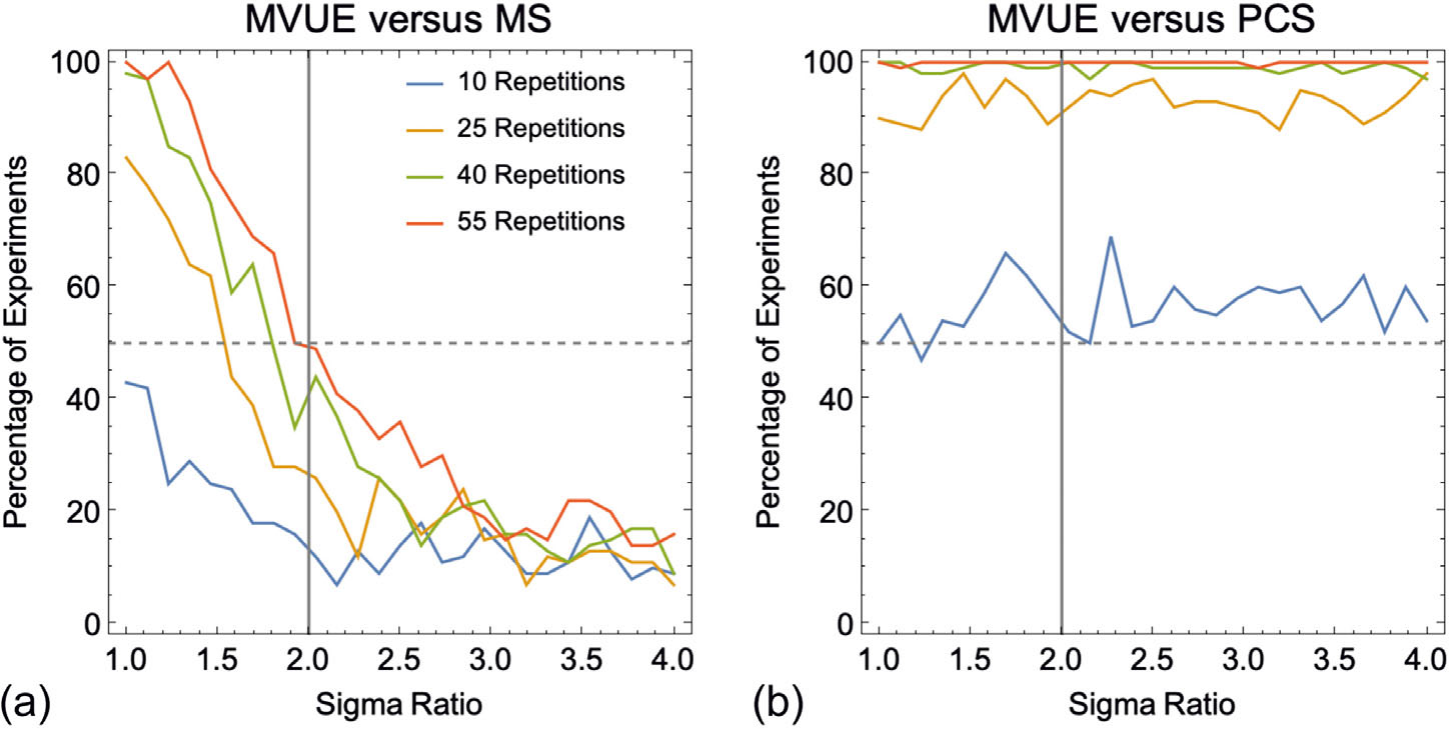
Plots the percentage of experiments in which the sigmas of the Cumulative Gaussian functions fit to a simulated population of four MVUE observers could be statistically distinguished from the experimentally derived prediction of (**a**) MS and (**b**) PCS. The dashed grey line represents the point at which there is a 50% chance of distinguishing the data from the predictions. The vertical grey line shows the maximum recommended sigma ratio to use in cue integration experiments (Rohde et al., 2016).

Many of the experiments shown in Figure 4 contain an unrealistically high number of observers per experiment. Therefore, Figure 5 plots the results for both comparisons, for the simulated experiments with four observers (as in Ernst & Banks, 2002 and Hillis et al., 2004). The vertical grey line shows the maximum recommended sigma ratio to use in cue integration experiments (Rohde et al., 2016), whereas the dashed grey line shows the point at which there is a 50% chance of distinguishing models. It is clear that with a representative number of observers in a typical cue integration experiment, to have any reasonable chance of distinguishing MVUE and MS, one needs to collect a large amount of data per participant and very closely match cue reliabilities. Collecting 150 trials per function across four observers with a sigma ratio of 2 would result in an approximately 25% chance of distinguishing these models, suggesting that existing guidelines (Rohde et al., 2016) may need to be improved upon. In contrast, even with four observers, PCS can be well distinguished from MVUE, for all but the lowest data collection regime.

#### Group analysis: Integrated cues percept

Next, we examined the extent to which MVUE can be distinguished from MS and PCS based upon the predicted integrated cues percept when a discrepancy is experimentally introduced between cues (Young et al., 1993). With zero cue conflict the only differences in ${\hat{S}}_{A}$, ${\hat{S}}_{B},$ and ${\hat{S}}_{C}$ will be due to chance, so any statistical differences will represent “false positives” (see Supplementary Figures S3, S4). The false positive rate was approximately 16% for MS (for 10, 25, 40, and 55 repetitions per function, the percentages are 15.99%, 16.09%, 16.21%, and 15.83%) and approximately 14% for PCS (for 10, 25, 40, and 55 repetitions, the percentages are 13.62%, 13.69%, 13.82%, and 13.57%). The difference between the false positives for MS and PCS is due to the effect that the sigma of the simulated function has on the inferred mean of the function across participants. Although the mean and sigma of a Cumulative Gaussian functions are mathematically independent, our ability to infer these parameters by fitting psychometric functions to data is not.

**Figure 6.**
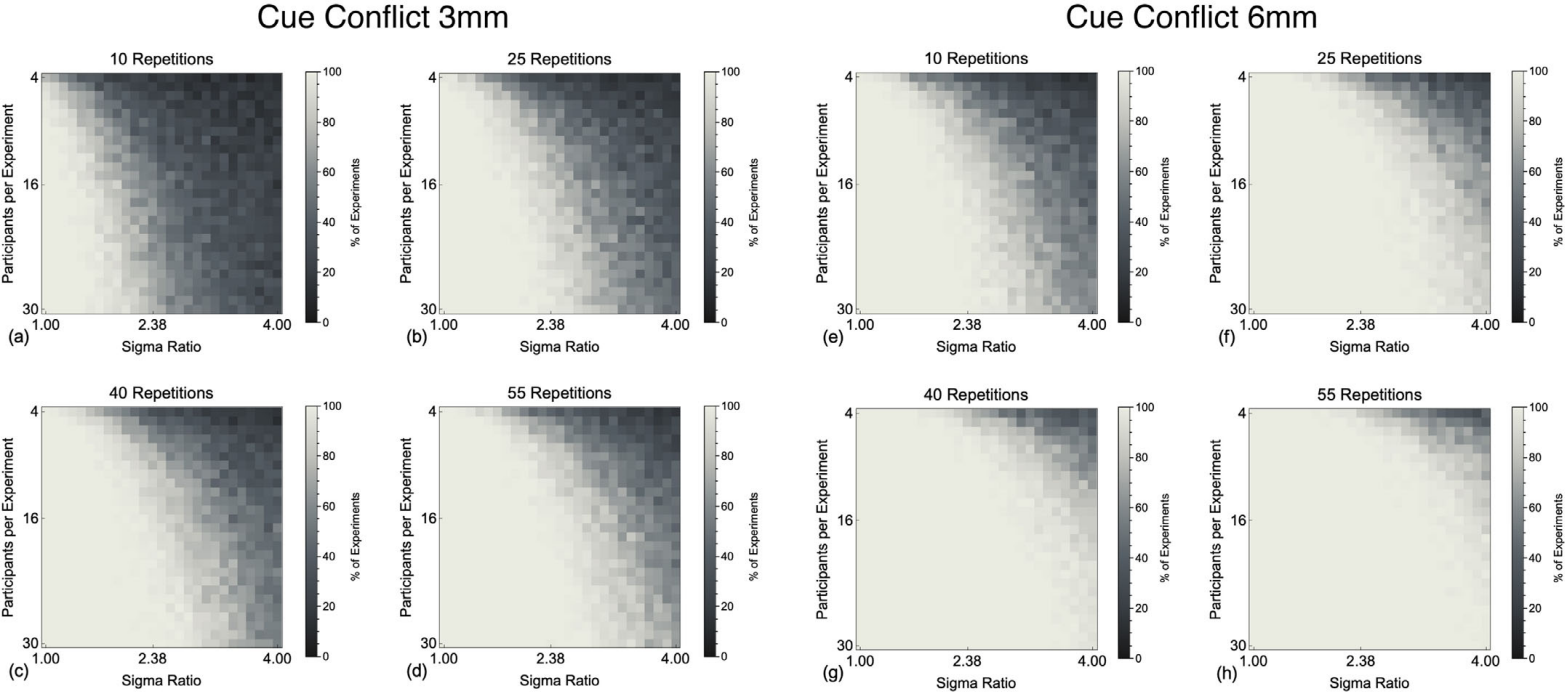
Shows the percentage of experiments in which the mean of the Cumulative Gaussian functions fit to our simulated population of MVUE observers could be statistically distinguished from the experimentally derived prediction of MS with experimental cue conflicts of 3 mm (**a–d**) and 6 mm (**e–h**). This is shown for **a** and **e** 10, **b** and **f** 25, **c** and **g** 40, and **d** and **h** 55, simulated trials per stimulus level on the psychometric function.

Figure 6 show the data for the 3 mm and 6 mm cue conflicts when comparing to the predictions of MS. As with distinguishing models based on the sigmas, the ability to distinguish between models is strongly affected by the relative reliability of the cues and the data collection regime. As would be expected, the probability of distinguishing between models is greater with a larger cue conflict. Due to PCS and MVUE providing identical predictions regardless of the experimental cue conflict, the only times a population of MVUE observers are distinguishable from the predictions of PCS again represent false positives (Supplementary Figures S5, S6).

**Figure 7.**
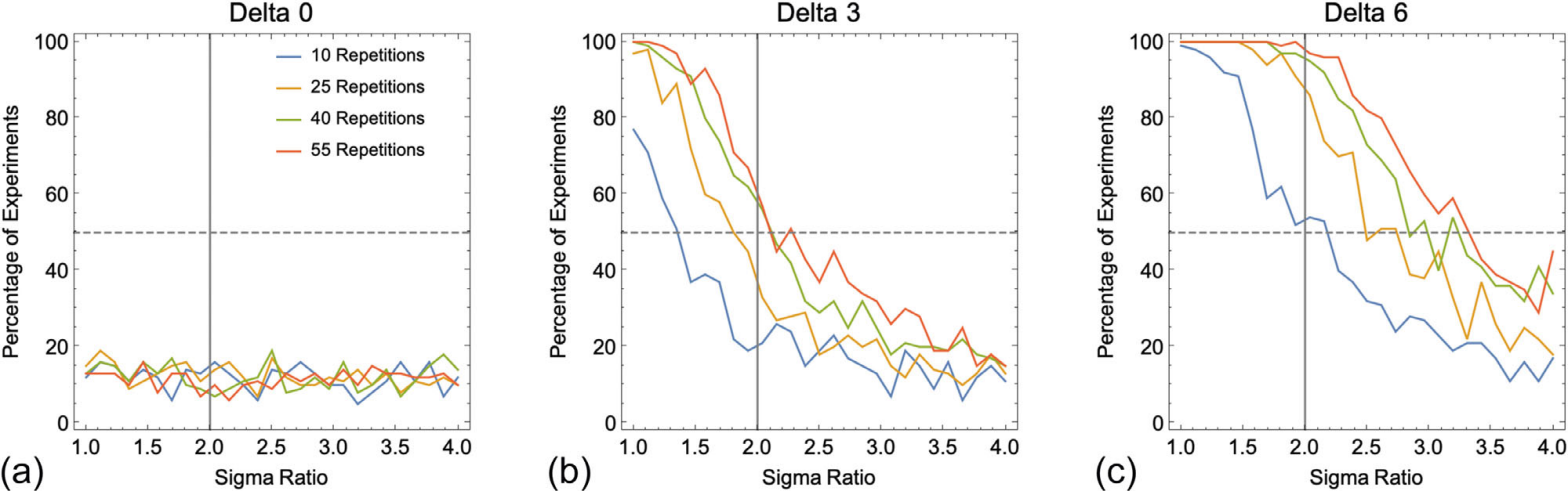
Plots the percentage of experiments in which the PSEs of the Cumulative Gaussian functions fit to a simulated population of four MVUE observers could be statistically distinguished from the experimentally derived prediction of MS. The dashed grey line represents the point at which there is a 50% chance of distinguishing the data from MS. The vertical grey line shows the maximum recommended sigma ratio to use in cue integration experiments (Rohde et al., 2016).

In Figure 7 we show the ability to experimentally distinguish between MVUE and MS based upon the integrated cues percept for just the simulated experiments with four observers (Ernst & Banks, 2002; Hillis et al., 2004). With no cue conflict (Delta of 0) the false positive rate is approximately 12% across all data collection regimes and sigma ratios. For both cue conflicts (Delta of 3 and 6 mm), the closer the reliability of cues is matched, and the more data collected, the better one can discriminate our population of MVUE observers from the predictions of MS. For a Delta of 3 mm (see Figure 7b), the ability to distinguish models rapidly drops off within the range of sigma ratios acceptable for a cue integration experiment (Rohde et al., 2016), such that with a sigma ratio of 3 and above, performance is comparable to that of the false positive rate (see Figure 7a). By comparison, with a Delta of 6 mm, within the range of sigma ratios acceptable for a cue integration experiment the ability to discriminate between models is good, with performance dropping substantially for only the most minimal data collection regime.

One of the most striking things about the analysis presented is just how rapid the drop-off is in an experimenter's ability to distinguish a population of MVUE observers from the predictions of MS, as the reliability of cues becomes unmatched. MVUE observers are easily distinguished from PCS in terms of the cue reliability, but impossible to distinguish based upon the integrated cues percept. MVUE observers can be more easily distinguished from MS based upon the integrated cues percept, but only dramatically so for larger cue conflicts. However, distinguishing models based upon the integrated cues percept alone is not sufficient to demonstrate that observers are behaving in accordance with MVUE (Rohde et al., 2016).

## Simulation set 2: Using variation across experimental observers to distinguish between models

In the second set of simulations, we examined the case where individual observers in an experiment had different relative cue reliabilities. This is a weaker form of testing MVUE as data collection can occur in regions of the parameter space which poorly distinguishes between models (see Figure 3), but it is more representative of a typical cue integration experiment where there may be variation in cue reliabilities across observers (Hillis et al., 2004; Scarfe & Hibbard, 2011) and properties of the stimuli may naturally (Hillis et al., 2004) or artificially (Ernst & Banks, 2002; Helbig & Ernst, 2007) be used to modulate the relative reliability of cues.

### Methods

For these simulations, we focused on comparing MVUE and MS, as these models can be distinguished based upon both the integrated cues percept and its precision. Observers were simulated as having access from two cues (${\hat{S}}_{A}$ and ${\hat{S}}_{B}$) from which to make an integrated cues perceptual estimate (${\hat{S}}_{C}$). These cues were in conflict such that *S_A_* = 55 + Δ/2 and *S_B_* = 55 − Δ/2 (in separate experiments, Δ was either 3 or 6 mm). ${\hat{S}}_{A}$ always had the same sigma σ_*A*_ = 4.86, which is approximately that of the haptic cue in Ernst and Banks (2002), whereas ${\hat{S}}_{B}$ had a randomly determined sigma of σ_*B*_ = σ_*A*_*r* where, consistent with the recommendations of Rhode et al. (2016), *r* ∈  [0.5,  2]. To select values with equal probability between these limits, for each observer we generated a random number x_*i*_ ∈  [ − 1,  1], and set $r=2^{x_{i}}$. Separate simulations were run with 4, 12, and 36 observers per simulated experiment, and for 10, 25, 40, and 55 trials per stimulus level. For each combination of (a) data collection regime, (b) number of observers per experiment, and (c) cue conflict (4  ×  3  ×  2), we simulated 1000 experiments (i.e. 32,000 experiments with 416,000 observers in total).

With a heterogenous population of observers the relationship between predicted and observed data are often compared using a linear regression analysis. For example, Burge, Girshick, and Banks (2010) examined the perception of slant from disparity and haptic cues and reported an R^2^ of 0.60 (significance not stated) for predicted versus observed integrated cues sensitivity. Knill and Saunders (2003) also examined the perception of slant, but from disparity and texture cues, and reported R^2^ values between around 0.15 and 0.46 (*p* < 0.05) for the predicted and observed cue weighting for different base slants. Svarverud et al. (2010) examined “texture-based” and “physical-based” cues to distance and reported R^2^ values of about 0.95 (*p* < 0.001) for predicted and observed cue weights. The median R^2^ value in these studies is 0.53 and in all instances the authors concluded that observers were combining cues optimally in accordance with MVUE. Following these studies, a regression analysis was adopted here.

For each experiment, the data from the population of observers behaving in accordance with *either* MVUE or MS were plotted against the predictions of each of the two candidate models. Data were fit with a first order polynomial by least squares and an R^2^ value for the fit of each model to the data calculated. Thus, there were four possible regression comparisons: (1) “MVUE versus MVUE” – predictions of MVUE, plotted against data from a population of observers behaving in accordance with MVUE; (2) “MS versus MS” – predictions of MS, plotted against the behavior of a population of observers behaving in accordance MS; (3) “MVUE versus MS” – predictions of the MVUE model, plotted against the data of a population of observers behaving in accordance with MS; and (4) “MS versus MVUE” – predictions of the MS model, plotted against the data of a population of observers behaving in accordance with MVUE. We will refer to (1) and (2) as “consistent” predicted and observed data as the simulated data and predictions are from the same model, and (3) and (4) as “inconsistent” predicted and observed data as the simulated data and predictions arise from different models.

**Figure 8.**
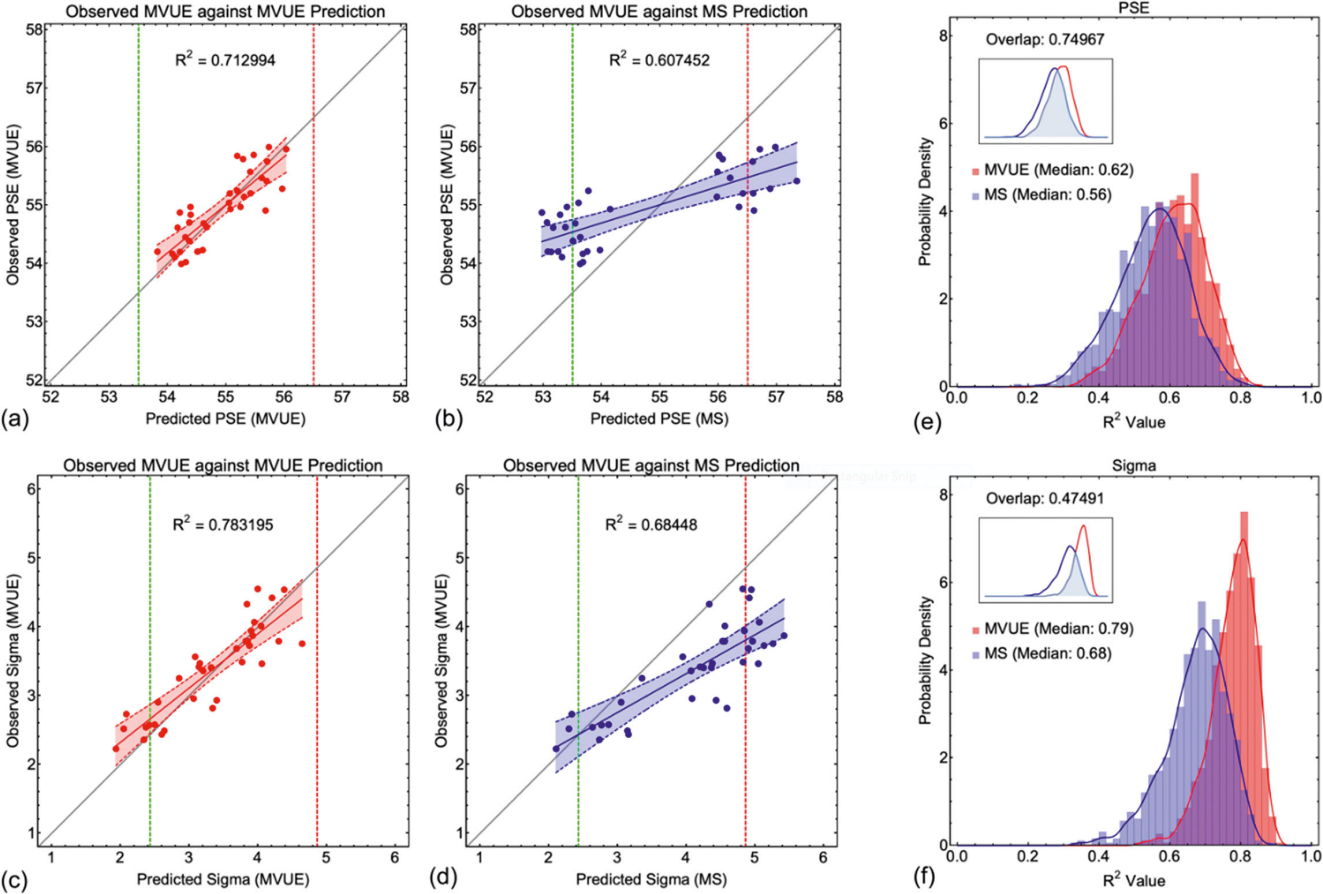
(**a**) through (**d**) show an example linear regression analysis where the data from 36 observers behaving in accordance with MVUE (with 55 samples per stimulus value and a delta of 3 mm) are plotted against the predictions of the two candidate models (MVUE **a** and **c**, and MS **b** and **d**), for both PSE **a** and **b** and Sigma **c** and **d**. The least squares first order polynomial is shown as the solid line, with the dashed lines and shaded region showing the 95% confidence bounds around the fit. In **a** and **b** the dashed red line shows the true underlying PSE for cue A, and the green dashed line shows the true underlying PSE for cue B. In **c** and **d** the red dashed line shows the (fixed) sigma for cue A, and the dashed green line the minimum possible sigma for cue B (which varied across simulated observers). (**e**) and (**f**) show the full distributions for the *R*^2^ value across all 1000 simulated experiments for **e** PSE and **f** sigma. Data are shown as bar histograms and as smoothed histograms (smoothed Gaussian kernel distribution; Equation 9). Red data are from MVUE observers plotted against the predictions of MVUE; blue data are from MVUE observers plotted against the predictions of MS. The median for each data set is shown in the graphs. The inset graph shows the overlap of the smoothed histograms (Equation 10). Note that the axes of the inset graphs are smaller to ensure clarity of the overlapping region.

A set of example data (PSE and sigma) from 36 observers behaving in accordance with MVUE (with 55 samples per stimulus value and a delta of 3 mm) is shown in Figure 8a to d for the “MVUE versus MVUE” and “MS versus MVUE” comparisons. This example represents the upper limit of observers and data collection in a typical cue combination experiment (Kingdom & Prins, 2016; Rohde et al., 2016). Figure 8a and b plot the PSE data from the MVUE observers against the experimentally derived predictions of the two candidate models, with the green and red dashed lines show the true underlying PSE for each cue. Figure 8c and d plot the observed sigma data from the MVUE observers against the experimentally derived predictions of the two candidate models, here, the dashed red line shows the fixed sigma of cue A and the green dashed line the minimum possible sigma for cue B.

What is most striking from this example is that the observed R^2^ values for both PSE's and sigmas are directly comparable to those found in the literature (and even better) regardless of whether the data from a population of MVUE observers were fitted with a regression against the predictions of either MVUE or MS. Figure 8e and f shows histograms of the observed R^2^ values for the same example, but across all 1000 simulated experiments. The raw histograms are shown overlaid with smooth kernel distributions, given by

(9) $${\hat{F}}_{X}\left(x\right)=\frac{1}{nh}\sum_{i=1}^{n}K\left(\frac{x-x_{i}}{h}\right)$$

Here,K is a Gaussian kernel function, x_*i*_ ∈  [0,  1] (i.e. the domain of the R^2^ value is 0 to 1), and ${\hat{F}}_{X}$ is the estimate of the unknown probability density function *F_x_*. The key parameter of interest is the extent to which these distributions overlap, as this determines the extent to which an R^2^ value from fitting predicted to observer data can be used to distinguish between candidate models of cue integration. The overlap of two smooth kernel distributions ${\hat{F}}_{X}$ and ${\hat{F}}_{Y}$ can be estimated via numerical integration (Pastore & Calcagni, 2019)

(10) $$\hat{\eta }\left(X,Y\right)=\int_{1}^{0}min\left({\hat{F}}_{X}\left(z\right),{\hat{F}}_{Y}\left(z\right)\right)dz$$

Numerically the overlap value lays between 0 (no overlap) and 1 (full overlap). This is shown inset in Figures 8e and f. As can be seen there is substantial overlap in the distribution of R^2^ values, especially so for the predicted and observed PSEs.

**Figure 9.**
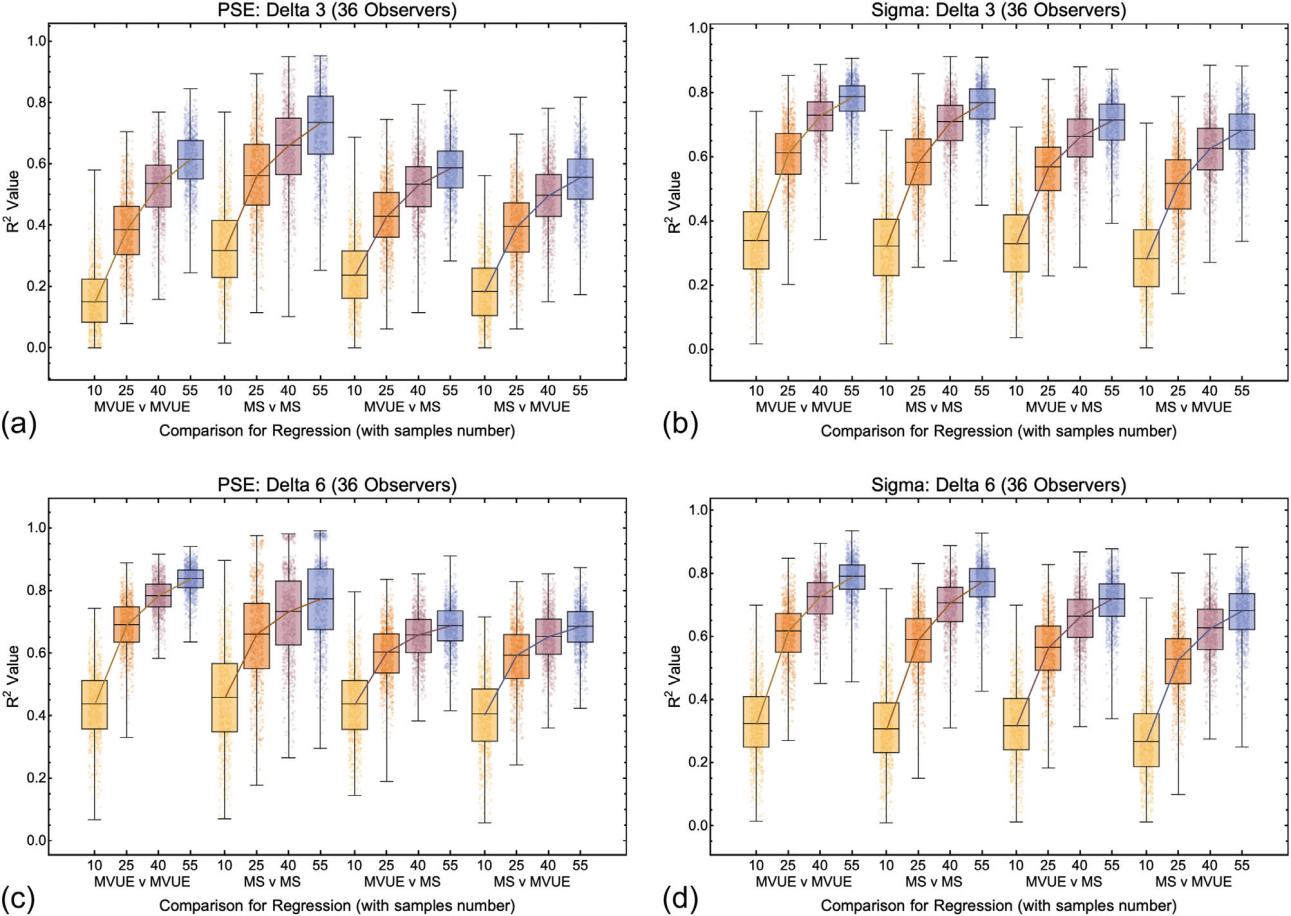
Box and whisker plots showing the distribution of *R*^2^ values for all conditions and comparisons in which there were 36 simulated observers per experiment for the 3 mm (**a** and **b**) and 6 mm (**c** and **d**) cue conflict (delta) conditions. The central box line shows the median (also shown as a line connecting the boxes), the limits of the boxes show the 25% and 75% quantiles and the limits of the bars (whiskers) show the maximum and minimum values. Also shown are all 1000 datapoints per condition (dots). For increased clarity the dots have been randomly jittered laterally.

**Figure 10.**
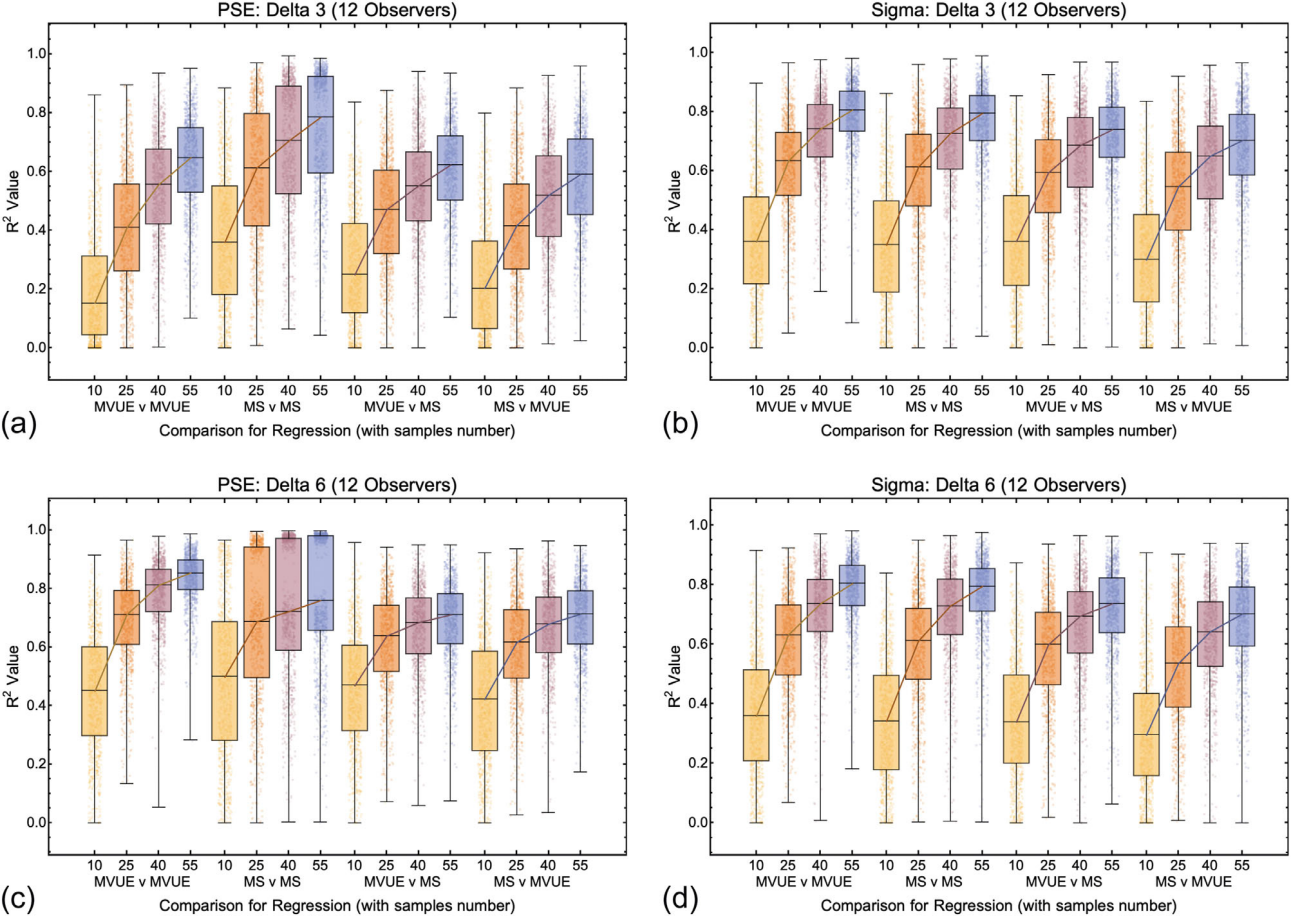
Box and whisker plots showing the distribution of *R*^2^ values for all conditions and comparisons in which there were 12 simulated observers per experiment for the 3 mm (**a** and **b**) and 6 mm (**c** and **d**) cue conflict (delta) conditions. The format is the same as Figure 9.

**Figure 11.**
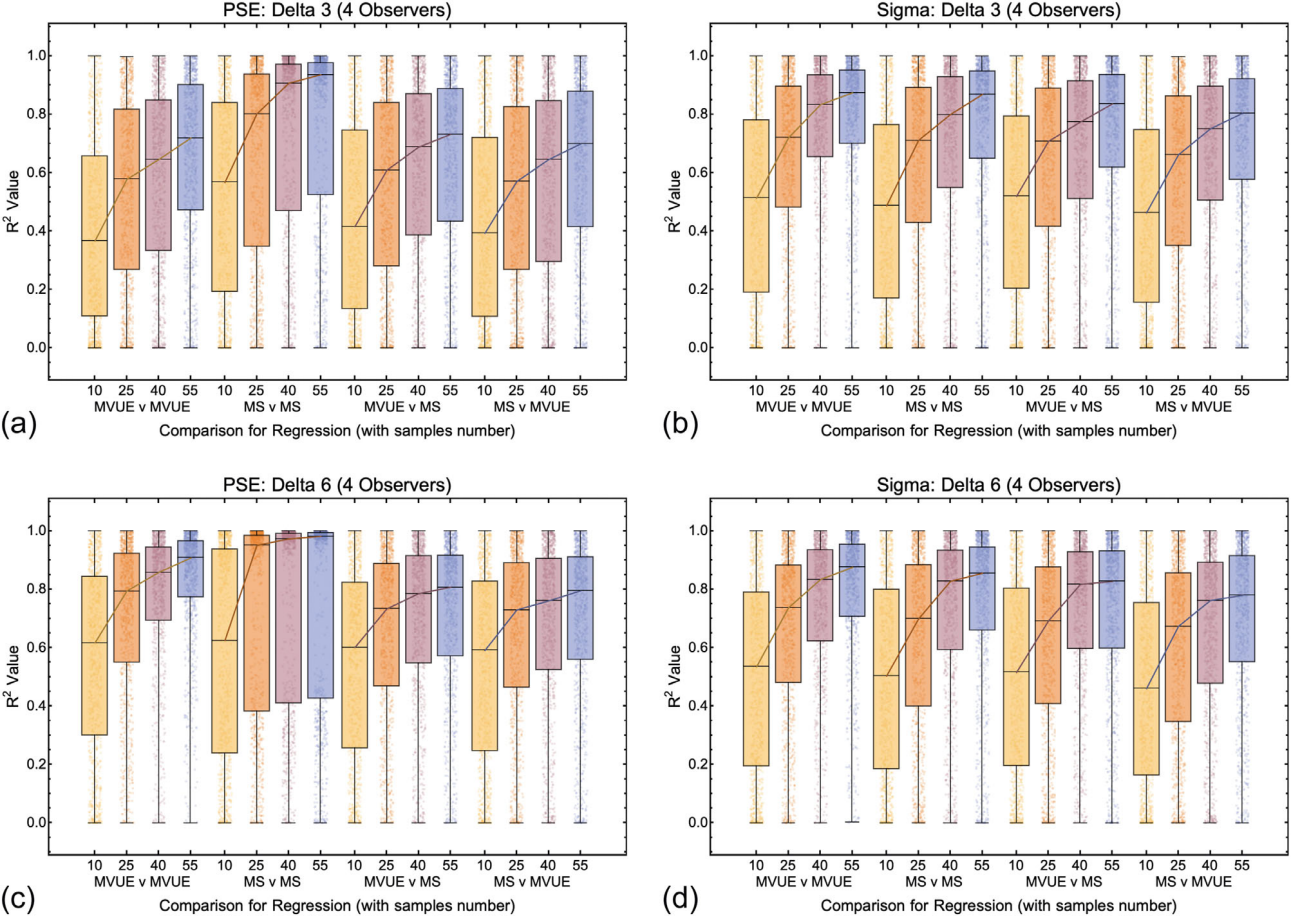
Box and whisker plots showing the distribution of *R*^2^ values for all conditions and comparisons in which there were four simulated observers per experiment for the 3 mm (**a** and **b**) and 6 mm (**c** and **d**) cue conflict (delta) conditions. The format is the same as Figures 10 and 11.

Data across all comparisons for both PSE and sigma are shown in Figures 9, 10, and 11. As one would expect, with more data collected per function and more observers per experiment the *R*^2^ values improve, with a maximal median of approximately 0.7 to 0.8. Problematically, this pattern is present regardless of whether one is plotting consistent predicted and observed data (MVUE versus MVUE and MS versus MS), or inconsistent predicted and observed data (MVUE versus MS and MS versus MVUE). Across all plots, there is the large overlap in the distributions of *R*^2^ values when plotting “consistent” and “inconsistent” predicted and observed data. With fewer observers per experiment (4 and 12 versus 36) the overlap increases greatly, to the extent that with four observers per experiment the data have near complete overlap.

**Figure 12.**
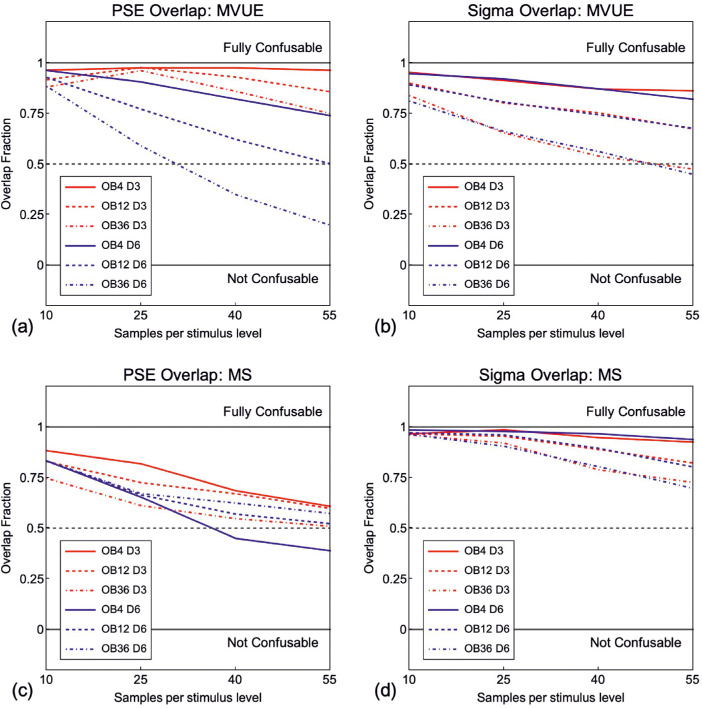
Overlap of the smooth kernel distributions of R^2^ value values produced from fitting a first order polynomial to observed data from observers behaving in accordance with MVUE against the experimentally derived predictions of MVUE and MS (PSE in (**a**) and Sigma in (**b**)), and a set of observers behaving in accordance with MS against the experimentally derived predictions of MVUE and MS (PSE in (**c**) and Sigma in (**d**)). Red lines are for the 3 mm cue conflict and blue lines are for the 6 mm cue conflict. “OB” is the number of observers in each simulated experiment and “D” the magnitude of the cue conflict in mm. An overlap value of 1 (upper solid grey line) means that the distributions (examples shown in Figures 8e and f) completely overlap and are “fully confusable” and overlap of 0 means that the distributions do not overlap at all and are thus “not confusable.” The dashed grey line shows the case where the distributions overlap by 50%.

Figure 12 ^13^ shows the overlap (Equation 10) for the distributions where a population of observers behaving in accordance with MVUE or MS were compared to the experimentally derived predictions of MVUE and MS. As expected, (1) the distribution overlap decreases with increasing amounts of data collected per function, (2) for the PSE distributions, the distribution overlap is less with a Δ of 6 mm versus 3 mm, and (3) the delta magnitude has no effect on the overlap of the sigma distributions. Problematically the distribution overlap is greater than 50% for virtually all conditions. This strongly questions one's ability to use *R*^2^ to assess the extent to which a set of data are consistent with the predictions of MVUE. The precise amount of quantitative overlap acceptable for an experiment would be a judgment on the part of the experimenter.

**Figure 13.**
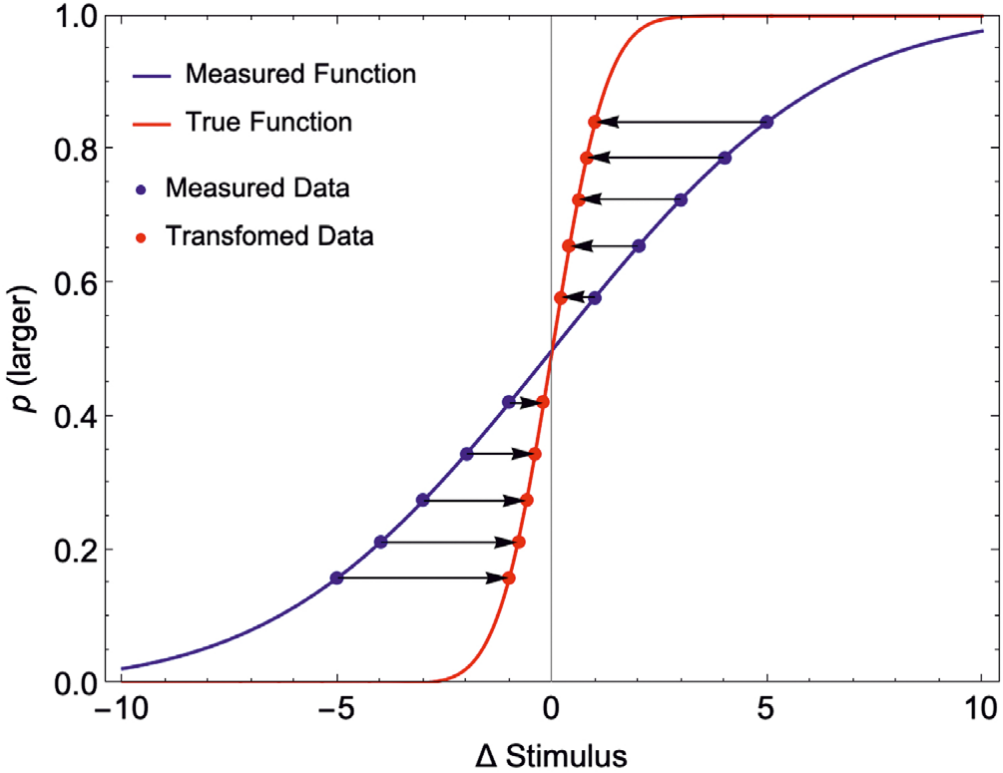
An experimenter presents a range of stimuli Δ*S_A_* and for each of these measures the probability of a “larger” response (“Measured Data,” shown as blue points). This is done in the presence of a conflicting cue, *S_N_*, which signals no change across intervals. For this visual example, σ_*A*_ = 1 and σ_*N*_ = 0.5, therefore *w_A_* = 0.2 and *w_N_* = 0.8. The experimentally measured points are consistent with a measured psychometric function (blue Cumulative Gaussian function (given by Equation 16)). This function has a standard deviation ${\hat{\sigma }}_{A}=\frac{\sigma_{A}}{w_{A}}=5$. In reality, each stimulus Δ*S_A_*(*i*) is in fact cue conflict stimuli Δ*S_C_*(*i*) (given by Equation 11), thus the data should be shifted along the x-axis toward Δ*S_A_* = 0 (black by arrows) to accurately plot the function. These shifted points (“Transformed Data,” shown as red points, Equation 12) are consistent with the true underlying psychometric function for the cue *S_A_* (red Cumulative Gaussian function (given by Equation 15)). This function is steeper than the (measured) blue function because for a measured p(larger), the Δ*S_A_* was in fact smaller than the experimenter had planned (due to the cue conflict).

An additional problem is that the *R*^2^ statistic that experimenters report does not measure the deviation of the data from the predictions of a cue integration model (even though it is often stated in this way), rather, the *R*^2^ statistic gives a measure of the fit of the polynomial. The predicted values of a cue integration model could be off by any arbitrary amount or have the opposite relationship between predictions and data, and experimenters could still obtain an *R*^2^ close to 1. Thus, a regression analysis negates one of the key benefits of MVUE (and other cue integration models), which is the ability to predict the absolute value of the integrated cues percept and its reliability and then compare this to that observed experimentally. Tests do exist to determine whether the intercept and slope differ from predicted model values, but these are rarely reported and are definitively not shown by the *R*^2^ statistic alone.

## Discussion

In any area of science, it is the job of a scientist to design experiments which can best distinguish between alternative models of the underlying phenomena. Unfortunately, in the area of cue integration, this is rarely done. There are a wide range of competing models for how human observers might integrate information from sensory cues (Beierholm et al., 2009; Jones, 2016; Körding et al., 2007; Mamassian, Landy, & Maloney, 2002; Trommershauser et al., 2011), but in many instances the results of an experiment are simply visually inspected relative to the predictions of the experimenters preferred model (Negen et al., 2018; Rohde et al., 2016). This is problematic due to the small benefit accrued by models such as MVUE, the highly correlated predictions provided by alternative candidate models, and the fundamental misconceptions researchers have about how error bars relate to statistical significance (Belia et al., 2005; Cumming et al., 2007).

Although the numerous assumptions the MVUE model (and others) are known, these are rarely tested, instead experimenters typically assume that the assumptions are met and claim support for MVUE, often in the absence of statistical analysis and/or sufficient model comparison. The present paper aimed to draw attention to the assumptions of MVUE and to introduce a principled method by which to determine the probability with which a population of observers behaving in accordance with one model of cue integration can be experimentally distinguished from the predictions of alternative models. This showed that the experimental approach taken in many studies results in a poor ability to distinguish between alternative models (and thus the claim support for MVUE). At all decision points the simulations were designed to be (1) consistent with published guidelines stating how to test models of cue integration (Rohde et al., 2016), (2) consistent with the existing literature (Ernst & Banks, 2002), and (3) consistent with best practice as regard to experimental methods (Fründ et al., 2011; Kingdom & Prins, 2016; Prins, 2012, 2013; Rohde et al., 2016; Wichmann & Hill, 2001a, 2001b).

Additionally, many of the nuisance parameters which would impede an experimenter's ability to distinguish between models were not simulated. For example, for our simulated observers there was (1) statistical independence between trials, with no learning or boredom effects (Fründ et al., 2011), (2) a known generative function underlying behavior (Kingdom & Prins, 2016; Murray & Morgenstern, 2010), (3) no perceptual bias (Scarfe & Hibbard, 2011), (4) stimulus values for the psychometric function were centered on the true mean of the psychometric function, (5) simulated observers exhibited no lapses (Prins, 2012; Wichmann & Hill, 2001a, 2001b), (6) the simulated data were not contaminated by the effect of decisional (or other sources of) noise (Hillis et al., 2004), (7) cues were statistically independent from one another (Oruc et al., 2003), and (8) there were no conflicting sources of sensory information (Watt et al., 2005). As a result, the simulations presented are highly likely to overestimate one's ability to experimentally distinguish between models. These nuisance factors are known problems across all types of behavioral experiment, so are likely to be present to some extent in most studies. As a result, experimenters have designed experiments to eliminate them as best as possible, or where this is not possible, examined the effect they could have on the data (see Hillis et al., 2004; Oruc et al., 2003; Watt et al., 2005).

### Controlling for the effects of conflicting cues when measuring “single cue” sensitivities

A grounding assumption of the cue integration literature is that there exist separable sources of sensory information which provide independent perceptual estimates about properties of the world (Ernst & Bulthoff, 2004). In practice, it rapidly becomes apparent just how difficult it is to experimentally isolate sensory cues and to eliminate alternate cues which are not of interest (Watt et al., 2005; Zabulis & Backus, 2004). In many instances, it remains possible that observers are utilizing sensory cues that the experimenter was not intending to be available (Ho, Landy, & Maloney, 2006; Saunders & Chen, 2015; Todd, 2015; Todd et al., 2010). Even more problematically, some experiments measure “single cue” sensitivities in the presence of a known conflicting sensory cue held constant (Murphy, Ban, & Welchman, 2013; Svarverud et al., 2010). Here, we examine the consequences of this.

Let's assume that an experimenter is using a two-interval forced choice experiment to measure the sensitivity of a cue *S_A_* for judgments of size. On each trial, in one interval, the experiment presents a “standard” stimulus and in the other interval a “comparison” stimulus, the difference between these being Δ*S_A_*. The observer must signal in which interval the “larger” stimulus was presented. Next, let's assume that this is done in the presence of a conflicting “nuisance” cue, *S_N_*, which is constant and signals that the stimulus is unchanged across intervals. This means that the “single cue” stimulus is in fact an integrated cues stimulus and can be described as

(11) $$\begin{array}{c} \Delta S_{c}=w_{A}\Delta S_{A}+w_{N}S_{N}\; \end{array}$$

For each stimulus value Δ*S_c_*(*i*), the experimenter measures $p\left(''larger“|\Delta S_{c}\left(i\right)\right)$ and (with the assumption that the “standard” and “comparison” stimuli can be represented by Gaussian probability density functions) maps out a psychometric function by plotting $p\left(''larger“|\Delta S_{c}\left(i\right)\right)$ against Δ*S_A_*(*i*), then fits a Cumulative Gaussian to the data (blue data and function in Figure 13). Clearly, the experimenter will incorrectly estimate σ_*A*_ from this fitted function. More specifically, they will overestimate σ_*A*_ because each stimulus that they present is in fact an attenuated version of that which they intended (i.e., Δ*S_c_*(*i*) < Δ*S_A_*(*i*)). The extent to which the experimenter misestimates σ_*A*_ will be a function of *w_N_* (the weight given to the nuisance cue *S_N_*, which is signally no change across intervals). As σ_*N*_ → ∞, the weight given to the nuisance cue will approach zero (*w_N_* → 0) and σ_*A*_ will be estimated accurately. However, for any non-infinite value of σ_*N*_, the experimenter will misestimate σ_*A*_.

In effect, what one needs to do is “warp” the x-axis of the measured psychometric function such that one is plotting $p\left(''larger“\right)$ against Δ*S_c_*(*i*) instead of Δ*S_A_*(*i*) (red data and function in Figure 13). To determine this “warping,” we can ask, what scale factor, *k*, would we need to apply to Δ*S_A_* such that in all cases Δ*S_c_* = Δ*S_A_*. Given *w_N_* = 1 − *w_A_*, we can write this as

(12) $$\begin{array}{c} \;\;\;\;\;\;\;\;\;\;\;\;\;\Delta S_{A}=\Delta S_{c}=w_{A}\left(S_{N}+\left(\Delta S_{A}*k\right)\right)+\left(1-w_{A}\right)S_{N}\;\;\; \end{array}$$

Recognizing that *S_N_* = 0 and solving for *k*, we get

(13) $$k=\frac{1}{w_{A}}$$

Intuitively we can see that this makes sense, as when *w_A_* = 1, no scaling is required to combat the attenuation caused by *S_N_*, because it receives zero weight, however, as soon as *w_A_* < 1, scaling is needed (i.e. *k* > 1). Next, we can ask, given the true value of σ_*A*_, what would be our estimate, ${\hat{\sigma }}_{A}$, of this be in the presence of the conflicting nuisance cue. To do this, we recognize that for a probability density function of a random variable *X* distributed according to *F_X_*(*x*), the probability density function of a variable *Y* = *g*(*X*) is also a random variable. If *g* is differentiable and g:R→R is a monotonic function, we can then use a change of variables to transform between probability density functions.

(14) $$F_{Y}\left(y\right)=F_{X}\left(x\right)\left|\frac{dx}{dy}\right|$$

Here, (*x*) = *g*^−1^(*y*) and the support of *Y* is *g*(*x*) with the support of *X* being *x* (Blitzstein & Hwang, 2015). For our example, the Gaussian probability density function representing our cue *S_A_* (red function in Figure 13) can be written as

(15) $$F_{X}\left(x\right)=\frac{1}{\sigma_{A}\sqrt{2\pi }}e^{-\frac{{\left(\mu -x\right)}^{2}}{2\sigma_{A}^{2}}}$$

This function has a mean of µ and standard deviation of σ_*A*_. From Equation 13, using the transform x*k, a change of variables gives

(16) $$F_{Y}\left(y\right)=\frac{w_{A}}{\sigma_{A}\sqrt{2\pi }}e^{-\frac{{\left(\mu -w_{A}*x\right)}^{2}}{2\sigma_{A}^{2}}}$$

This represents our experimentally inferred probability density function for cue *S_A_* (blue function in Figure 13). The standard deviation of *F_Y_*(*y*) is given by

(17) $${\sigma_{A}}^{'}=\frac{\sigma_{A}}{w_{A}}$$

When weighting is given to the nuisance cue, *w_A_* < 1, we overestimate the sigma of the underlying estimator, ${\sigma_{A}}^{'}$>σ_*A*_.

We can now determine the consequences this has for measuring the relative reliability of cues, which is the key variable needed for testing MVUE. Let's say we have two cues *S_A_* and *S_B_* with standard deviations of σ_*A*_ and σ_*B*_ signalling a property of interest, *S*. We measure “single cue” sensitivity functions for each cue while holding the other cue constant. Because $1/\sigma_{A}^{2}+1/\sigma_{B}^{2}$ is a constant, *c*, the weights given to each cue are $w_{A}=\frac{1}{c*\sigma_{A}^{2\;}}$ and $w_{B}=\frac{1}{c*\sigma_{B}^{2}}$, and given in Equation 17, our experimental estimates of the true underlying standard deviations are given by ${\hat{\sigma }}_{A}=c*\sigma_{A}^{3}$ and ${\hat{\sigma }}_{B}=c*\sigma_{B}^{3}$. These are each larger than the true underlying values as they have been measured in the presence of a cue signally no change (see Figure 13). The ratio of these estimates is given by

(18) $$\frac{{\hat{\sigma }}_{A}}{{\hat{\sigma }}_{B}}=\frac{\sigma_{A}^{3}}{\sigma_{B}^{3}}$$

Thus, the ratio of the underlying sigma's, which is the property we wish to estimate, is given by

(19) $$\frac{\sigma_{A}}{\sigma_{B}}=\sqrt[{3}]{\frac{{\hat{\sigma }}_{A}}{{\hat{\sigma }}_{B}}}$$

Therefore, if we infer from our experiment that σ_*A*_/σ_*B*_ = 1/27 the true sigma ratio is in fact 1/3 and we experimentally misestimate σ_*A*_/σ_*B*_ by a factor of approximately 9. Studies which have measured the reliability of cues in the presence of a secondarily constant conflicting cue (e.g. Murphy et al., 2013 and Svarverud et al., 2010), will therefore have significantly overestimated the true cue relative reliabilities. As such, the data in these studies cannot be used to accurately test MVUE, without some form of correction. This analysis shows the critical importance of being able to isolate singles cues satisfactorily, or if one is not able to, correct for their influence when inferring relative cue reliabilities.

## Conclusion

The simplicity of the MVUE equations for cue integration is deceptive. A model's simplicity is generally correlated with the number of assumptions it makes about the underlying phenomena. With more assumptions and a simpler model, there is a greater chance that the assumptions of the model will not be met. This will impact an experimenter's ability to accurately test the predictions of the model. Even if one can be satisfied that the assumptions of MVUE hold in an experimental situation, MVUE provides correlated predictions with many other cue integration models (Arnold et al., 2019; Beierholm et al., 2009; Jones, 2016). Here we considered two such models, MS and PCS. It was shown that even when adopting published criteria describing how to best test the predictions of MVUE (Rohde et al., 2016), it could be very difficult to experimentally disambiguate among MVUE, MS, and PCS. The analysis presented is only scratching the surface, as there are many ways in which sensory cues could be integrated (Jones, 2016), some of which may be even more difficult to disambiguate from MVUE.

Many studies claiming to support MVUE fail to consider alternative models satisfactorily, sample areas of the parameter space which poorly distinguish between competing models, and provide no statistical analysis related to the fit of MVUE to the data, or the relative fit of other alternative models. This questions the ability of these studies to conclude that sensory cues are integrated in accordance with MVUE. Although one could interpret the results presented here in a pessimistic fashion, the opposite is true. The results show clear, simple, and computationally attainable ways in which experimenters can correctly measure the variables needed to test models of cue integration and determine the probability with which a population of observers behaving in accordance with one model of sensory cue integration can be experimentally distinguished from the predictions of alternative models. Furthermore, it can be argued that the focus should not be upon attempting to prove that cues are integrated “optimally” based upon some criterion, but rather to simply focus on the factors that influence how cues are integrated (Rosas & Wichmann, 2011).

## Supplementary Material

Supplement 1
