# Supplementary material for: Experimentally disambiguating models of sensory cue integration
Source: J Vis. 2022 Jan 12;22(1):5. doi: 10.1167/jov.22.1.5 (PMC8762719; doi:10.1167/jov.22.1.5)
Supplement: Supplement 1 [file jovi-22-1-5_s001.pdf]

S1: Estimating data from Ernst and Banks (2002)

Single cues sensitivities used for the simulations reported were estimated from Figure 3d of Ernst and Banks (2002). In order to gain estimates of the single cue sensitivities we viewed Figure 3d (as a pdf file) on a 4K computer monitor, so that the graph filled the majority of the screen. We then took the pixel coordinates of (1) the data points, (2) the minimum and maximum error bar position for each data-point and (3) the minimum and maximum values on the *x*- and *y*-axes. We were then able to compute the relative position of each data point (and error bar) in pixel coordinates on the *x*- and *y*-axis and covert these to the units shown in the graph by using the measured correspondence between pixel coordinates and axis units. Visual comparison of Figure 2 of the present paper and Figure 3d of Ernst and Banks shows that close correspondence achieved.

There was some inconsistently in Ernst and Banks (2002) as to how a "threshold" or "discrimination threshold" was defined. On page 430 the authors state, "The discrimination threshold is defined as the difference between the point of subjective equality (PSE) and the height of the comparison stimulus when it is judged taller than the standard stimulus 84% of the time". However, on page 431 the authors state "… T_H and T_V are the haptic and visual thresholds (84% points in Fig. 3a)". It is the first definition which is consistent with the mathematics i.e., the difference between the PSE and 84% point of the function being equal to the sigma of the fitted Cumulative Gaussian function.

Therefore, we cross checked our thresholds estimated from Figure 3d of Ernst and Banks (2002), with the thresholds calculated from the integrated cues functions in Figure 3b of Ernst and Banks (2002). Thresholds from Figure 3b were taken to be the difference between the point of subjective equality (PSE) and the 84% point on the function. When compared to the thresholds estimated from Figure 3d the difference in estimates was very small (average across data points of 0.23). We were therefore happy that definition of threshold was that of page 430 and that we had accurately estimated the thresholds and understood their relationship to the properties of the psychometric functions reported in the paper. Note: that for the purposes of the present paper all that was needed is an approximation of the exact values.

S2: Example functions and goodness of fit

Figure S2a shows the true underlying functions for the minimum, maximum and base (middle) sigma values used in the current study as well as the stimulus levels at which the functions were sampled. As can be seen, consistent with Ernst and Banks (2002) Figure 3a, all functions

straddle high and low performance levels needed for well fit functions (Wichmann & Hill, 2001a, 2001b). Figures S2b-e show examples of how these functions were sampled with our four sampling regimes (10, 25, 40 and 55 trials per stimulus level), with the maximum likelihood best fit functions and goodness of fit (see below) values shown in the legend. We have only shown these for just the $\Delta = 0$ case, as for all delta values used the sampling range was shifted so as to be centred on the true mean of the underlying function. As is clear, for all sampling regimes the data are well fit by the Cumulative Gaussian functions.

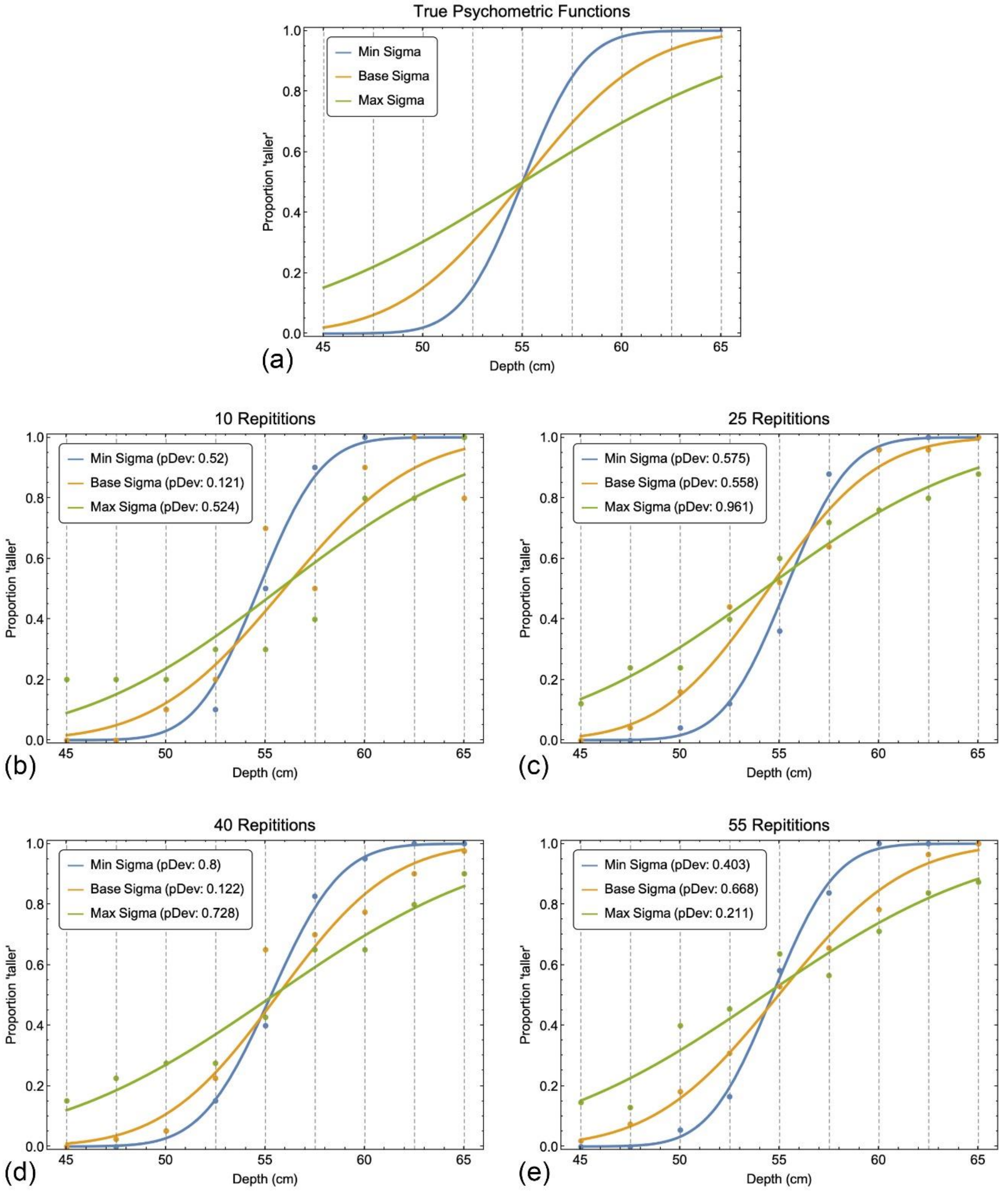

***Figure S2**: (a) shows the underlying "true" psychometric functions for the minimum, maximum and base (middle) sigma values used in the paper. The dashed vertical grey lines show the nine stimulus values at which these functions were sampled. (b) through (e) show examples of how the functions were sampled through simulation and fit with psychometric functions for the four data collection regimes used throughout the paper (10, 25, 40, and 55 repetitions per stimulus level). Inset in each graph is the goodness of fit value, pDev. This represents the probability with which the experimental data produced a higher likelihood ratio than that of the stimulated experiments. If this is greater than 0.05, the function is considered to fit the data well. See accompanying text for details.*

Within the cue integration literature, the goodness of fit of a function and the criteria upon which a fit is considered unacceptable is rarely if ever stated (for example Ernst & Banks, 2002; Helbig & Ernst, 2007; Hillis et al., 2002). Thus, it is impossible to tell if a goodness of fit test was performed, and if one was, which test which was used, and the criteria adopted for rejecting a fitted function. Given that the fit of data to the MVUE model is normally assessed by eye, it is likely that this is also the case for the fit of individual psychometric functions (Kingdom & Prins, 2016). The Palamedes toolbox (Prins & Kingdom, 2009) used in the present study implements a bootstrapped likelihood ratio test to assess the goodness of fit of a psychometric function. The logic of the test is as follows (Kingdom & Prins, 2016).

As detailed in the main text, when fitting a psychometric function to some data the experimenter assumes: (1) the observer does not improve or degrade at the task they are performing over time, (2) each perceptual judgement an observer makes is statistically independent of all others, and (3) performance of the observer can be well characterised by the psychometric function that the experimenter is choosing to fit to the data. These assumptions combined can be referred to as the "target model". The validity of the target model can be assessed by comparing it to a "saturated model" which only assumes (1) and (2). Thus, in the saturated model, the probability of response for one stimulus level is completely independent on the probability of response for any other stimulus level i.e., no psychometric function is assumed.

The target model is "nested" under the saturated model, as it is a single specific case of the saturated model. Thus, the likelihood associated with the fit of the target model can never produce a better fit than that of the less restrictive saturated model. For a given set of data one can calculate the likelihood ratio (likelihood of the target model / likelihood of the saturated model) which will, by definition, be less than or equal to 1. It will only be equal to one if the target and saturated models provide as good a fit as one another. The likelihood ratio test, implemented in the Palamedes Toolbox, simulates a set of experiments through a bootstrap procedure where the simulated observer is behaving in accordance with the more restrictive target model. The simulated data is fit twice, once under the assumptions of the

target model and once under the assumptions of the saturated model, and a likelihood ratio calculated. The probability with which the experimental data produces a higher likelihood ratio than that of the stimulated experiments is calculated (*pDev* in Figure S2). If this probability is less than 0.05% the goodness of fit is deemed poor. As with any *p*-value, the 0.05% cut-off is a completely arbitrary convention (Kingdom & Prins, 2016). Thus, some experimenters may adopt this and others not. This mirrors the open discussion about the use of *p*-values for general statistical analysis.

For the present study, it was computationally unfeasible to run a bootstrapped likelihood ratio test for each of the ~15.3 million simulated functions (even when using MATLAB's Parallel processing toolbox to spread the computational load over the 8-Core Intel Core i9 available to the author this would have taken ~1-2 months of constant processing). Nevertheless, we wanted to assess the extent to which the maximum likelihood fit functions would in general be considered well fit. Therefore, for the maximum and minimum cue sigma value used in the paper (i.e. shallowest and steepest psychometric functions), we simulated data for 1000 observers, fit Cumulative Gaussian psychometric functions to the data (as described in the main text) and assessed the goodness of fit using the bootstrapped likelihood ratio test (1000 bootstraps). We did this for our four sampling regimes: 10, 25, 40 and 55 trials per stimulus level.

Based upon the 0.05% criteria for a cut-off between well and poorly fit function (*pDev* in Figure S2), virtually all functions would have been classed as well fit, regardless of data collection regime of the slope of the underlying function (Table T2; overall average 94.95%). As would be expected, this was true for all Delta levels. This is because the sampling range was always centred on the true mean of the function, so the values for Delta 0, 3 and 6 in Table T2 are effectively replications of one another. This confirms across 24000 fitted functions what can be seen in the example functions of Figure S2 i.e. that the data are well fit by the psychometric functions. We can therefore be satisfied that the around 94.95% of all functions reported in the paper would have been classed as well fit based on this criteria. See also the criteria adopted for rejecting psychometric functions discussed in the main body of the text.

**Sigma / Trials**	**Delta 0**	**Delta 3**	**Delta 6**
**Min 10**	93.8%	95.5%	95.4%
**Max 10**	95.5%	95.2%	95.5%
**Min 25**	96.2%	94.5%	94.6%
**Max 25**	95.6%	96.7%	95.4%
**Min 40**	95.7%	95.5%	94.1%
**Max 40**	94.7%	95%	95.2%
**Min 55**	95.5%	94.1%	94.4%

**Max 55**	94.7%	93.7%	94.7%
**Mean Value**	**94.91%**	**95.03%**	**94.91%**

***Table T2**: Shows the percentage of psychometric functions which would be classified as well fit based upon the bootstrapped likelihood ratio test described in the main text. The percentage of well fit functions is shown for the minimum and maximum sigma used in the simulations of the paper, and for each combination of trials per stimulus value on the psychometric function and cue conflict level (cue delta in mm).*

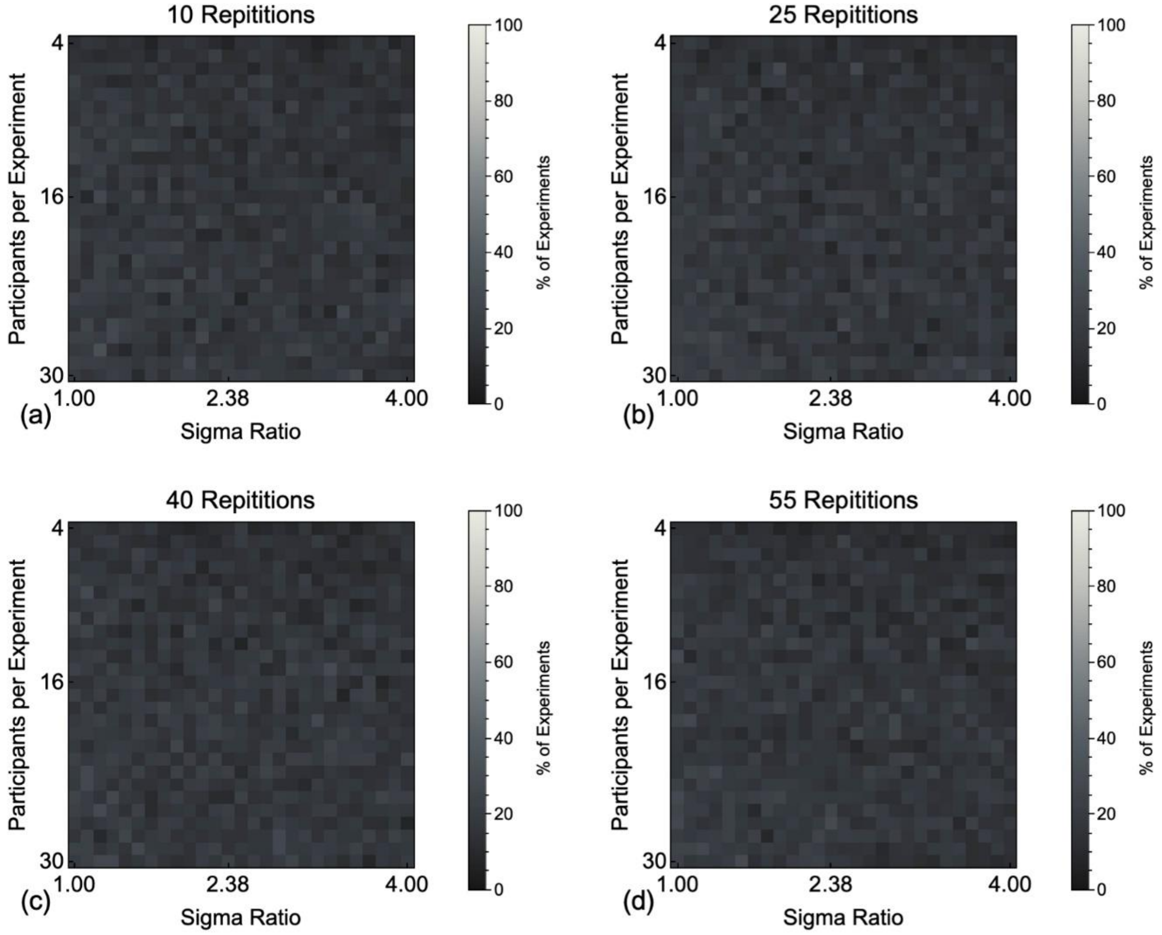

***Figure S3:** Shows the percentage of experiments in which the mean of the Cumulative Gaussian functions fit to our simulated population of MVUE observers could be statistically distinguished from the experimentally derived prediction of MS, when there is zero cue conflict. Each pixel in the image shows this percentage as calculated across 100 simulated experiments, of a given sigma ratio and number of participants. The four panes show this for (a) 10, (b) 25, (c) 40 and (d) 55, simulated trials per stimulus level on the psychometric function.*

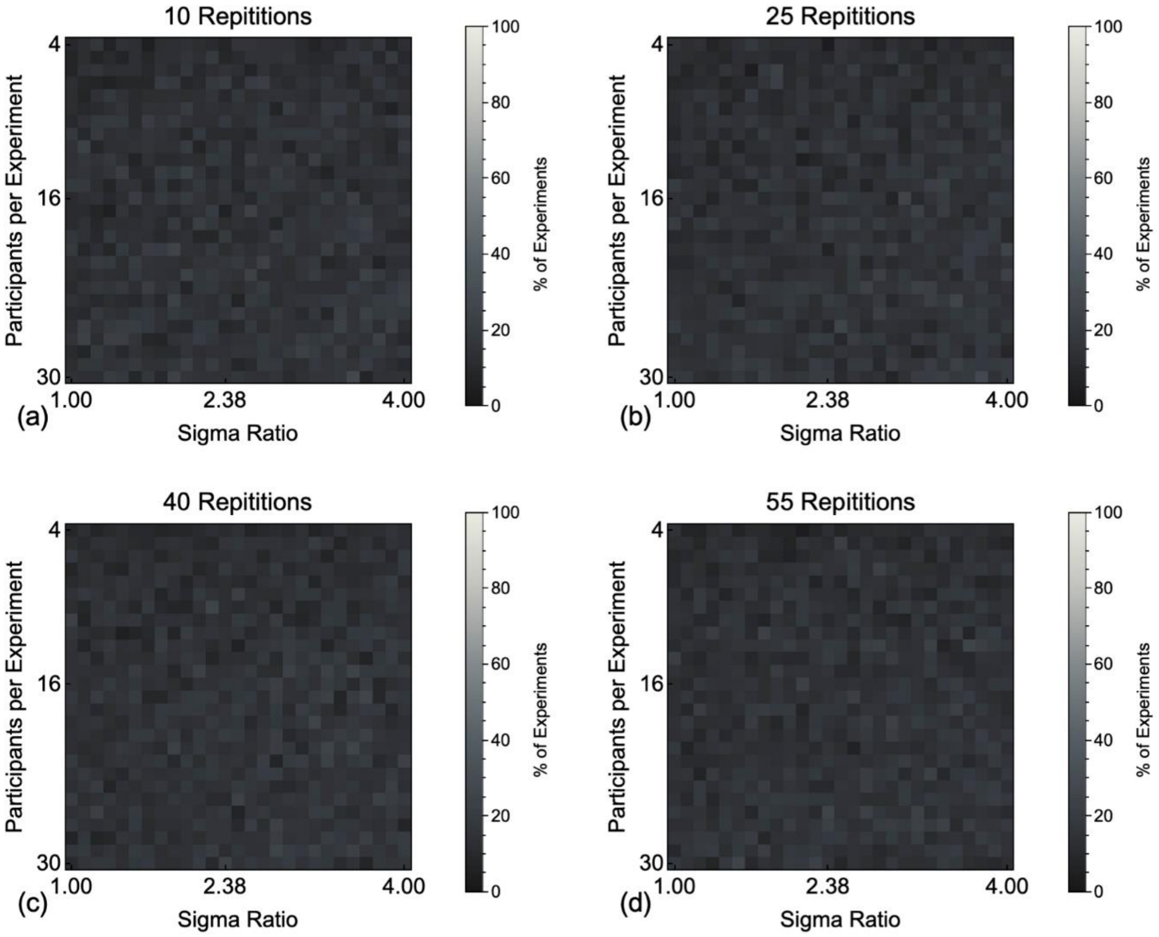

Figure S4: *Shows the percentage of experiments in which the mean of the Cumulative Gaussian functions fit to our simulated population of MVUE observers could be statistically distinguished from the experimentally derived prediction of PCS, when there is zero cue conflict. Each pixel in the image shows this percentage as calculated across 100 simulated experiments, of a given sigma ratio and number of participants. The four panes show this for (a) 10, (b) 25, (c) 40 and (d) 55, simulated trials per stimulus level on the psychometric function.*

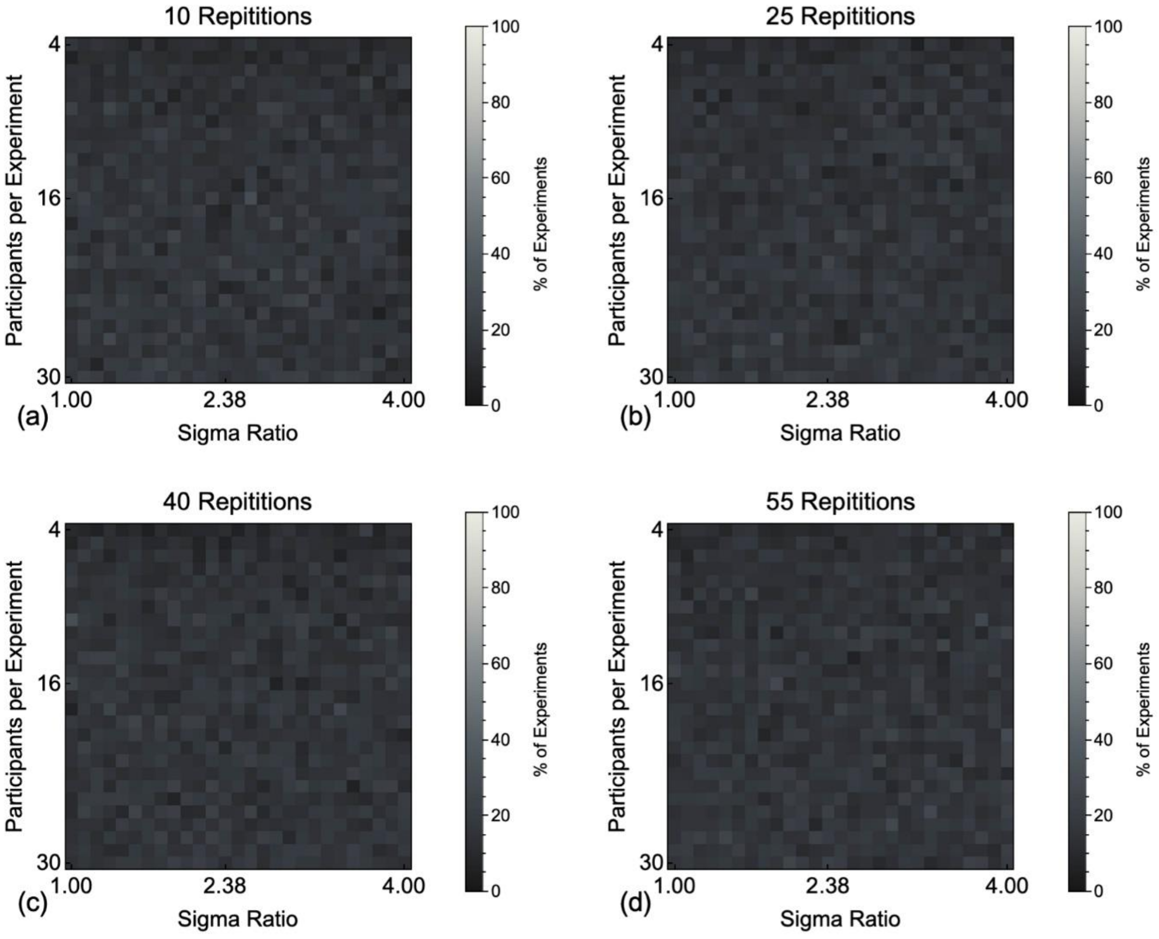

Figure S5: *Shows the percentage of experiments in which the mean of the Cumulative Gaussian functions fit to our simulated population of MVUE observers could be statistically distinguished from the experimentally derived prediction of PCS with an experimental cue conflict of 3mm. Each pixel in the image shows this percentage as calculated across 100 simulated experiments, of a given sigma ratio and number of participants. The four panes show this for (a) 10, (b) 25, (c) 40 and (d) 55, simulated trials per stimulus level on the psychometric function.*

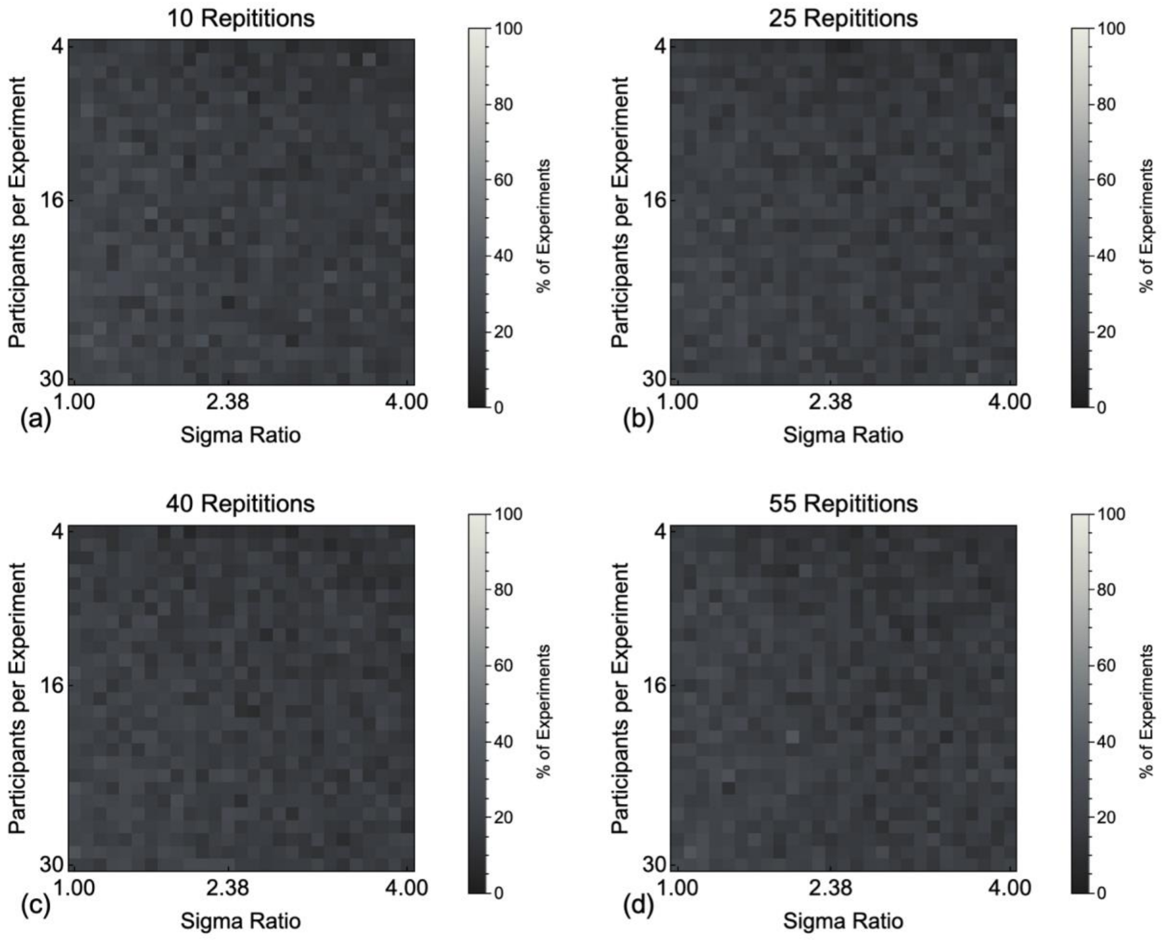

Figure S6: *Shows the percentage of experiments in which the mean of the Cumulative Gaussian functions fit to our simulated population of MVUE observers could be statistically distinguished from the experimentally derived prediction of PCS with an experimental cue conflict of 6mm. Each pixel in the image shows this percentage as calculated across 100 simulated experiments, of a given sigma ratio and number of participants. The four panes show this for (a) 10, (b) 25, (c) 40 and (d) 55, simulated trials per stimulus level on the psychometric function.*

Ernst, M. O., & Banks, M. S. (2002). Humans integrate visual and haptic information in a statistically optimal fashion. *Nature*, *415*(6870), 429-433. https://doi.org/10.1038/415429a

Helbig, H. B., & Ernst, M. O. (2007). Optimal integration of shape information from vision and touch. *Exp Brain Res*, *179*(4), 595-606. https://doi.org/10.1007/s00221-006-0814-y

Hillis, J. M., Ernst, M. O., Banks, M. S., & Landy, M. S. (2002). Combining sensory information: Mandatory fusion within, but not between, senses. *Science*, *298*(5598), 1627-1630. <Go to ISI>://000179361600051

Kingdom, F. A. A., & Prins, N. (2016). *Psychophysics: A Practical Introduction.* (2nd ed.). Academic Press.

Prins, N., & Kingdom, F. A. A. (2009). *Palamedes: Matlab routines for analyzing psychophysical data.* http://www.palamedestoolbox.org.
Wichmann, F. A., & Hill, N. J. (2001a). The psychometric function: I. Fitting, sampling, and goodness of fit. *Percept Psychophys*, *63*(8), 1293-1313. https://www.ncbi.nlm.nih.gov/pubmed/11800458
Wichmann, F. A., & Hill, N. J. (2001b). The psychometric function: II. Bootstrap-based confidence intervals and sampling. *Percept Psychophys*, *63*(8), 1314-1329. https://www.ncbi.nlm.nih.gov/pubmed/11800459
